# Nanomedicine Strategies to Target Tumor-Associated Macrophages

**DOI:** 10.3390/ijms18050979

**Published:** 2017-05-04

**Authors:** Karin Binnemars-Postma, Gert Storm, Jai Prakash

**Affiliations:** 1 Targeted Therapeutics, Biomaterials Science and Technology, MIRA Institute for Biomedical Technology and Technical Medicine, University of Twente, 7522NB Enschede, The Netherlands; k.a.postma@utwente.nl (K.B.-P.); g.storm@uu.nl (G.S.); 2 Department of Pharmaceutics, Utrecht University, 3584CS Utrecht, The Netherlands

**Keywords:** nanoparticles, macrophages, tumor-associated macrophages, passive targeting, active targeting, cancer

## Abstract

In recent years, the influence of the tumor microenvironment (TME) on cancer progression has been better understood. Macrophages, one of the most important cell types in the TME, exist in different subtypes, each of which has a different function. While classically activated M1 macrophages are involved in inflammatory and malignant processes, activated M2 macrophages are more involved in the wound-healing processes occurring in tumors. Tumor-associated macrophages (TAM) display M2 macrophage characteristics and support tumor growth and metastasis by matrix remodeling, neo-angiogenesis, and suppressing local immunity. Due to their detrimental role in tumor growth and metastasis, selective targeting of TAM for the treatment of cancer may prove to be beneficial in the treatment of cancer. Due to the plastic nature of macrophages, their activities may be altered to inhibit tumor growth. In this review, we will discuss the therapeutic options for the modulation and targeting of TAM. Different therapeutic strategies to deplete, inhibit recruitment of, or re-educate TAM will be discussed. Current strategies for the targeting of TAM using nanomedicine are reviewed. Passive targeting using different nanoparticle systems is described. Since TAM display a number of upregulated surface proteins compared to non-TAM, specific targeting using targeting ligands coupled to nanoparticles is discussed in detail.

## 1. Introduction

As long ago as 1863, Rudolf Virchow noticed leukocytic infiltration in neoplastic regions; he was the first to link inflammation to cancer [1]. During tumor progression, not only are the malignant cells characterized by genetic mutations, but the tumor stroma, consisting of the extracellular matrix (ECM) and cells within it, is also known to play a crucial role in this process [2,3]. Due to the resemblance of tumor stroma to the granulation tissue formed during wound healing, tumors were described as “wounds that never heal” by Hal Dvorak [4]. In recent years, our understanding of the cross-talk between malignant cells and the tumor stroma has greatly increased. The inflammatory process caused by the tumor cells and the surrounding stroma has been shown to promote tumor growth and progression [2,3,5]. Since the tumor stroma cells are more genetically stable than malignant cells, they represent promising targets for therapeutic intervention. This review provides a summary of state-of-the-art technologies for the treatment and nanoparticle-based targeting of tumor-associated macrophages (TAM).

## 2. Stromal Inflammation

A major drive for tumor progression is the inflammatory process that can originate from intrinsic or extrinsic pathways. In the intrinsic pathway, genetic mutations cause oncogene activation, while the extrinsic pathway includes external stimuli, such as infection or chemical insult. Chronic inflammation creates an environment that increases malignant transformation, either by causing DNA damage and impeding DNA repair, or by inducing mutations and proliferation of already mutated cells [6]. These inflammatory processes can take place at specific sites of the body, for example in the colon, where inflammatory bowel disease increases the risk of developing malignancies. Studies have suggested that inhibition of the inflammatory process may reduce the risk of developing cancer [7,8,9].

The course of immune cell infiltration and activation of stromal cells leading to cancer-related inflammation is intricate. Many different cell types (mainly macrophages, dendritic cells, neutrophils, fibroblasts, and T-cells), and tumor cells themselves, play crucial roles in regulating this process. In solid tumors, tumor cells produce transcription factors like NF-κB, signal transducer and activator of transcription 3 (STAT3), and hypoxia-inducible factor 1α (HIF-1α), all of which have a profound effect on the recruitment, differentiation, and maturation of infiltrating leukocytes [10]. Moreover, tumor cells produce chemokines (such as chemokine C–C motif 2 (CCL2), -7 and -8) and cytokines, which attract inflammatory cells to the tumor site [11]. Macrophages, whether resident or recruited, due to the local cytokine environment, acquire the TAM phenotype, stimulating tumor survival, growth, and metastasis [10,11,12,13]. Macrophage and TAM biology will be discussed in more detail below.

Only in the last few years has the role of neutrophils in tumor progression been illustrated, although the relationship between circulating neutrophil numbers and poor prognosis has been established for years [14]. There are different phenotypes for neutrophils: the antitumor N1 phenotype and the protumoral N2 phenotype [15]. During early tumor development, N2 neutrophils promote angiogenesis and subsequently support tumor growth and invasion by remodeling the ECM [15,16,17,18].

## 3. Macrophage Polarization

Macrophages are commonly known phagocytic cells that can display various kinds of functional activities depending on their polarization state. They are one of the most plastic cell types known so far. Resident macrophages in tissues can display a high range of phenotypes, such as Kupffer cells in the liver, microglia in brain, alveolar macrophages in lungs, and peritoneal macrophages in the gut [19,20,21]. During inflammation, infiltrated monocytes derived from the bloodstream transform into mature macrophages in specific tissues. These macrophages may transform into either classically activated (M1) macrophages, which have pro-inflammatory properties in general, or become alternatively activated (M2) macrophages, which show anti-inflammatory and tumor-promoting capabilities [13]. The polarization state is largely dependent on the cues they receive from the local microenvironment [22]. The M1 and M2 nomenclature mirrors that of T helper cell type 1 and T helper cell type 2 (Th1 and Th2), since these cells differentially produce interferon-γ (IFN-γ) and intereukin (IL)-4, cytokines that are important in the polarization of M1 and M2 types, respectively [20]. Macrophages are often classified as either M1 or M2 cells, but these represent extremes in a system of continuous functional states. They usually display a phenotype somewhere in between M1 or M2, and for this reason they are more correctly described as either M1-like or M2-like macrophages. Here, for the sake of simplicity we will refer to them as M1 or M2 macrophages. Below, we discuss the M1 and M2 phenotypes in more detail.

### 3.1. M1 Macrophages

Classically activated or M1 macrophages are involved in the resolution of bacterial infection and exert anti-tumor activities. These macrophages display pattern recognition receptors, such as toll-like receptors (TLRs), which are able to recognize bacterial patterns, including lipopolysaccharide (LPS), muramyl peptide, and lipoteichoic acid [23,24]. Macrophages can become polarized by these bacterial products, but also by cytokines secreted by Th1 cells (e.g., IFN-γ). Following activation, M1 macrophages stimulate the adaptive immune response by releasing the pro-inflammatory cytokines IL-1, IL-6, IL-12, IL-23, and tumor necrosis factor-α (TNF-α). M1 macrophages can also be recognized by their different metabolism, e.g., arginine is metabolized by inducible nitric oxide synthase (iNOS), producing nitric oxide (NO), which has cytotoxic effects [12,25]. There are no characteristic receptors that are able to mark macrophages as the M1 phenotype, but they do overexpress certain receptors, e.g., cluster of differentiation (CD) 16, CD86, CD80, IL-1R I, major histocompatibility complex class II (MHC II), TLR2, and TLR4 [26]. They are sometimes characterized by the cytokines which they secrete, marking them by their IL-10^low^, IL-12^high^ profile [27]. M1 macrophages, either activated by bacterial products or IFN-γ, display high antigen presentation and high pro-inflammatory cytokine (e.g., IL-12 and IL-23) production, which stimulates the Th1 response. Moreover, they are able to produce high amounts of toxic intermediates (nitric oxide (NO) and reactive oxygen species (ROS)), which allows M1 macrophages to be potent effector cells in killing microbes and tumor cells [13,28,29,30].

### 3.2. M2 Macrophages

Unlike M1 macrophages, alternatively activated or M2 macrophages are involved in the wound healing process, allergies, and the killing of parasites, and they display pro-tumoral activities. Th2 cells elicit the stimuli (e.g., IL-4, IL-13) that are needed to polarize macrophages towards M2 phenotype. Upon differentiation, M2 cells display many receptors that are rather specific, e.g., decoy IL-1R II receptor, the macrophage scavenging receptor I (CD204), the mannose receptor (CD206), and the hemoglobin scavenger receptor (CD163) [26]. Also, because of the presence of arginase in these cells, arginine is not metabolized towards NO but to ornithine and polyamines, which are necessary for collagen synthesis and cellular proliferation [27]. M2 cells generally have a low production of pro-inflammatory cytokines, while they produce large amounts of IL-10 (IL-10^high^, IL-12^low^) [13,31,32].

Besides the normal M2 macrophages, there are also macrophages that have an “M2d state” following stimulation by immune complexes, LPS, or glucocorticosteroids [20,23]. These cells share some of the characteristics of M2 macrophages, but also produce cytokines such as IL-1 and IL-6, normally seen in M1 cells [20,23].

## 4. Tumor-Associated Macrophages

Tumor-associated macrophages (TAM) are key tumor stromal cell types and play a critical role in tumor survival, growth, and metastasis [10]. TAM may either originate from resident macrophages or are attracted from the bone marrow and spleen to the tumor site by the CCL2 (also known as monocyte chemoattractant protein-1 (MCP-1)). TAM are often compared to M2 macrophages. Indeed, they display characteristics of the M2 phenotype, sharing functions such as matrix remodeling, promoting angiogenesis, suppressing inflammation, and secreting growth factors [13]. Numerous investigations have shown a positive correlation between macrophage numbers and prognosis, both in human and murine malignancies [33,34,35]. The cytokine profile (macrophage colony stimulating factor (M-CSF), IL-4, IL-13, IL-10, Prostaglandin E2 (PGE2)) that is present in the tumor microenvironmentTME polarizes these macrophages and as a result they display pro-tumoral functions. Recently it has been shown that lactic acid, a byproduct of anaerobic glycolysis in tumor cells under hypoxic conditions, induces the gene expression of *Vegf* and *Arg-1* in TAM. This effect was mediated by HIF-1α. The lactate-induced expression of Arginase-1 was shown to induce tumor growth [36]. Following acquisition of the TAM phenotype, these macrophages display a number of functions that generally lead to pro-tumoral effects, as detailed below.

### 4.1. Suppression of Adaptive Immunity

Suppression of the adaptive immune system is achieved by TAM through various mechanisms. TAM lack the proper ability for antigen presentation, making them unsuitable for eliciting an immune response by other immune cells. TAM also secrete anti-inflammatory cytokines such as IL-10 and TGF-β, while being unable to secrete the pro-inflammatory cytokine IL-12, a lack of which leads to induction of T-regulatory (Treg) cells. This in turn suppresses the activity of effector T-cells and other immune cells such as monocytes [13,37]. A possible regulator in these processes is STAT3, which is overactivated in TAM [38]. ROS have also been shown to play a critical role in macrophage differentiation. Recently, it has been shown that pretreatment of monocytes with the antioxidant butylated hydroxyanisole inhibits polarization of monocytes towards the M2 type, but not the M1 type, suggesting that ROS are necessary for the polarization of monocytes towards the M2 type [39]. Following differentiation, monocytes produce superoxide (O^2−^), which is needed for the biphasic activation of the ERK pathway, a critical pathway in macrophage differentiation [39].

### 4.2. Matrix Remodeling, Tumor Invasion, and Metastasis

The prognosis of patients with high numbers of infiltrating macrophages in primary tumors is unfavorable: in triple negative breast cancer, TAM infiltration was quantified using immunohistochemical staining for CD68. High numbers of infiltrating macrophages are associated with a significantly higher risk of distant metastasis, as well as a decreased disease-free and overall survival [40]. In endometrial adenocarcinoma, the presence of TAM was associated with advanced disease staging, high tumor grade, increased lymph vessel density, lymphovascular space invasion, and lymph node metastasis [41]. There is mounting evidence that TAM play a critical role in tumor invasion and metastasis [12,42,43]. In the mouse mammary tumor virus-driven polyomavirus middle T antigen (MMTV–PyMT) model of mammary carcinogenesis, in macrophage-deficient mice, tumor progression and metastasis were significantly delayed [44].

One of the most important mechanisms of tumor cell migration, induced by macrophages, has been elucidated in the past: it was shown that in in vivo experiments macrophages and tumor cells were both able to migrate to stimuli specific for only one of the cell types (i.e., colony-stimulating factor-1 (CSF-1) for macrophages and epidermal growth factor (EGF) for tumor cells), indicating the existence of a paracrine loop, leading to a synergistic relationship between migration of both macrophages and tumor cells [45]. Indeed, tumor cells produce CSF-1, which induces migration of macrophages. Macrophages in turn, produce EGF, which leads to tumor cell migration [45,46]. An important biological barrier in invasion and metastasis is the basement membrane. During cancer progression, this membrane is degraded by proteolytic enzymes. Matrix metalloproteinases (MMPs) are a class of proteases that play an important role in this process. Macrophages are able to produce a variety of MMPs, such as MMP-1, -2, -7, -9, and -12 in a TNF-α-dependent manner [47]. The ECM also plays an important role in tumor cell invasion. During mammary gland development, macrophages have been shown to promote collagen fibrillogenesis [48]. Fibrillar collagen 1 facilitates the movement of macrophages and tumor cells, at up to 10 times the speed at which they would move through the tumor stroma [49]. As these collagenous fibrils anchor easily to blood vessels, tumor cells are guided towards them, making it possible for tumor cells to escape via the vasculature [43,49].

### 4.3. Neo-Angiogenesis

HIF play an important role in macrophage recruitment and angiogenesis. Due to rapid growth, tumors are prone to develop hypoxic areas. In an HIF-dependent manner, vascular endothelial growth factor (VEGF), C–X–C motif chemokine ligand-12 (CXCL12) and C–X–C chemokine receptor-4 (CXCR4) attract macrophages to these hypoxic areas [10]. This leads to the activation of a pro-angiogenic program: increased expression of VEGF, basic fibroblast growth factor (bFGF), and CXCL8, also regulated by HIF-1 and 2 [13]. Although macrophages themselves can be a source of VEGF, it is also released from the ECM by ECM degradation caused by macrophage-derived MMP-9 [50]. Casazza et al. showed that hypoxia-induced Sema3A acts as a chemo attractant for TAM through the neuropilin-1 (Nrp-1) and PlexinA1/PlexinA4 receptor complex. They demonstrated that Nrp-1 is downregulated in hypoxic areas, but stop signals were still produced via signaling by Sema3A through the PlexinA1/PlexinA4 complex. This resulted in trapping of the recruited TAM in the hypoxic tumor regions. Interestingly, deletion of the *Nrp-1* gene in macrophages resulted in the retention of macrophages in normoxic areas, subsequently leading to diminished pro-angiogenic and immunosuppressive functions. This study concluded that localization of macrophages, regulated by Sema3A/Nrp-1 signaling, plays an important role in their functional states [51]. In addition to degrading the basement membranes and ECM, TAM provide activated endothelial cells with an environment (via increased availability of VEGF and production of CXLC-1, -2, and -8), in which they can migrate, proliferate, and form new blood vessels [52,53,54].

## 5. Targeting Strategies and Active Agents

Proximity of TAM to the TME is necessary for TAM to maintain their tumor-promoting functions. Several strategies have been proposed in literature to reduce the tumor-promoting functions of TAM:

- Inhibiting macrophage recruitment;
- Reprogramming TAM towards a more anti-tumoral phenotype;
- Initiation of immune response;
- Blocking the tumor-promoting functions of TAM;
- Depletion of TAM.

### 5.1. Inhibiting Macrophage Recruitment

Inhibition of macrophage infiltration can be accomplished at two levels: (1) preventing chemo-attractants secreted by the tumor cells to recruit macrophages; or (2) blocking of macrophage surface receptors, thereby effectively preventing signal transduction or adhesion to be established.

CCL2 is involved in the recruitment of monocytes to the tumor site. Several small molecular inhibitors and antibodies against this protein have been used in various types of cancer. The small molecule inhibitor Bindarit has been reported to be effective in reducing tumor growth and macrophage recruitment in CCL2-positive melanoma [55]. Another approach is to block the receptor for CCL2, i.e., the C–C motif chemokine receptor 2 (CCR2). A small molecule inhibitor (RS102895) was found to be effective in inhibiting macrophage migration. In addition, two monoclonal antibodies (Carlumab and MLN1202) directed against CCL2/CCR2 are currently under investigation for their effectiveness in treating cancer [56,57,58]. Furthermore, an antibody against CD11b was used in a mouse squamous cell carcinoma xenograft model [59]. CD11b is the α-subunit of the CD18 integrin, which is expressed on both granulocytes and macrophages. This subunit has been shown to be involved in the adhesion, migration, and chemotaxis of myeloid cells [60]. In this study, the authors showed that, by administering CD11b antibodies, they inhibited the recruitment of myeloid cells into the xenografts and improved the response to irradiation [59]. A recent development for preventing macrophage recruitment is the use of inhibitors for the colony-stimulating factor receptor-1 (CSF-1R): PLX3397, BLZ945, and GW2580 [61,62,63,64]. PLX3397 showed reduction in macrophage infiltration in tumors in mouse models for neurofibroma, melanoma, gastrointestinal stromal tumors and malignant peripheral nerve sheath tumors, thereby reducing tumor growth [61,65,66,67]. The inhibitor BLZ945 was tested in a mouse model for glioblastoma multiforme. Surprisingly, TAMs were not depleted, but their survival was facilitated by IFN-γ and granulocyte macrophage colony stimulating factor (GM-CSF), while M2 markers decreased in surviving TAMs [64]. A third CSF-1R antagonist GW2580 was tested in combination therapy. Simultaneous treatment with gemcitabine augmented the effect of chemotherapy [63]. Next to small molecular inhibitors, the monoclonal antibody RG7155 has also been found to profoundly affect tumor macrophage populations. In a phase I clinical trial of patients suffering non-diffuse giant cell tumors, dose escalation studies have shown a compelling decline in CSF-1R^+^/CD163^+^ macrophages, which translated into clinical objective responses [68]. Currently, this antibody is investigated in phase 1a/1b clinical trials as monotherapy or in combination with other cancer immunotherapy agents [69].

### 5.2. Reprogramming and Blocking Tumor-Promoting Functions of Tumor-Associated Macrophages and Initiating Immune Response

Even though TAM exhibit tumor-promoting M2 characteristics, studies have shown that, depending on the cytokines present, they can be reprogrammed towards the tumoricidal M1-like phenotype [64,70]. Introducing Th1-cytokines or interfering in the transcription pathway leading to M2 macrophages are promising ways to modulate macrophage polarization. Another way for reprogramming is activation of the NF-κB pathway via the introduction of TLR-agonists [71]. These receptors play a major role in the innate immune system via pattern recognition of pathogens and inducing a Th1 response. In addition to introducing Th1 cytokines or ligands for TLRs, stimulating the immune response by activating co-stimulatory proteins such as CD40 has shown promising results [72,73,74]. Recently, a clinical trial using the fully humanized CD40 agonist antibody in combination with gemcitabine treatment in advanced pancreatic cancer was completed [72]. In this trial, the authors observed a partial clinical effect, the mechanism of which was later elucidated in a murine model for pancreatic cancer, where they found a modified macrophage phenotype, with increased MHC class II and CD86 expression [72]. In another study, CD40 agonist antibodies were combined with immunostimulatory CpG oligodeoxynucleotides in a model for murine multiple melanoma. Here, due to co-stimulation, macrophages were activated towards the M1 phenotype and a strong increase in IL-12 production was observed [75]. Indeed, restoring M1 macrophage functionality in tumors relieves the immunosuppressive environment and allows other effector immune cells to be recruited [76].

Other than monoclonal antibodies, Tasquinimod, a small molecule inhibitor of S100A9 and HDAC4, both important signaling molecules in the TME, used in the treatment of prostate cancer was investigated for its effects on TAM [77]. This inhibitor was found to increase the secreted amount of intra-tumoral IL-12, which resulted in a decrease of neo-vascularization and TAM infiltration as well as an increase in M1 macrophages. Furthermore, another study showed that a combination of Tasquinimod with an immunotherapy resulted in decreased M2-polarized macrophages and increased immune response [78]. Another molecule which inhibits macrophage polarization is 4-iodo-6-phenylpyrimidine (4-IPP), an inhibitor of macrophage migration inhibitory factor (MIF). MIF, both excreted by tumor cells and macrophages, has been linked to tumor promotion in paracrine and autocrine manners [79,80,81]. In melanoma-bearing mice, administration of 4-IPP led to attenuated TAM polarization, immunosuppression, neo-angiogenesis and melanoma outgrowth, offering MIF inhibition as an interesting target for further investigation [81].

In addition to preventing macrophage recruitment and reprogramming towards the tumoricidal phenotype, the prevention of M2 TAM formation has been investigated. One of the signaling pathways that, upon activation, causes M2 differentiation is the STAT3 pathway. The role of STAT3 in TAM as an important target for cancer immunotherapy was first described by Cheng et al. They showed that the disruption of the STAT3 pathway restored the responsiveness of immunotolerant T-cells from tumor-bearing mice [82]. Since then, many drugs have been used to inhibit the STAT3 pathway, including the clinically used kinase inhibitors sorafenib and sunitinib [83,84].

Another pathway involved in the differentiation towards M2 macrophages is the STAT6 pathway. Following binding of the M2-inducing cytokines IL-4 and IL-13 to their receptors, STAT6 becomes phosphorylated via Janus kinases (JAKs) and translocates to the nucleus, where it acts as a transcription factor [85]. Currently identified STAT6 inhibitors include AS1517499, TMC-264 and the active metabolite of Leflunomide (A771726) [71]. Binnemars-Postma et al. showed they could inhibit STAT6 phosphorylation and macrophage polarization towards the M2 phenotype using AS1517499. Tumor growth was significantly inhibited in the murine 4T1 mammary tumor model. Moreover, genetic markers for TAM infiltration (measured by *F4/80* and *YM-1*) and pro-metastatic markers (*matrix metalloproteinase-2* (*MMP-2*), *Periostin* and *CD34*) were significantly inhibited in the livers of treated mice, suggesting STAT6 is involved in the formation of the pre-metastatic niche [86].

### 5.3. Depletion of TAM

The most radical way of blocking TAM activity in tumors is to deplete them altogether. Currently, the most effective way of achieving this is by adding compounds that are toxic specifically to macrophages. Frequently used agents are bisphosphonates, including clodronate and zoledronic acid, but more recently trabectedin as well [71,87].

Bisphosphonates are classically used to inhibit osteoclast function and thus bone resorption in the treatment of osteoporosis, bone metastasis and Paget’s disease. Their mode of action is based upon the inhibition prenylation of proteins such as Ras, causing apoptotic cell death. Upon administration, bisphosphonates have a high binding affinity for hydroxyapatite and are therefore readily distributed to the bone, where they are taken up by the highly endocytic osteoclasts. In macrophages, bisphosphonates have been found to affect proliferation and motility and induce apoptosis [88]. Due to their extremely short half-life, most bisphosphonates by themselves do not reach sufficient tissue concentrations to exert any effect on macrophages [89]. However, when using clodronate-loaded liposomes, van Rooijen et al. found a transient decrease in spleen macrophages as soon as one day after injection [90]. In subsequent studies, the inhibition of tumor growth via macrophage depletion was established [91,92]. In a recent study using a murine xenograft model for cutaneous T-cell lymphoma, the administration of clodronate-loaded liposomes led to a reduction in tumor growth [93]. In the tumor tissue, the authors showed a decrease in pSTAT3 and total macrophage count, suggesting that clodronate-loaded liposomes caused a decrease in TAM and thereby a decrease in tumor cell growth [93]. Combinations of the various ways of targeting macrophages have also been investigated, using the combination of zoledronic acid with the previously mentioned STAT3 inhibitor sorafenib [94]. In a nude mouse xenograft model of hepatocellular carcinoma, the combination of drugs (rather than the individual drugs) was shown to significantly reduce tumor growth, metastasis, and angiogenesis [94]. More recently, nitrogen-containing bisphosphonates were found to be effective without the aid of a carrier. In this study, risedronate was able to bind to micro-calcifications, which were subsequently taken up by macrophages, but not by tumor cells [95].

Trabectedin, isolated from the sea squirt *Ecteinascidia turbinata*, is used in the treatment of soft tissue sarcoma, breast, and ovarian cancer [96]. This compound has been shown to induce apoptosis in macrophages as well. Its mechanism of action is complex and not fully understood. It is able to bind to DNA in the minor groove, bending it towards the major groove, severely influencing DNA structure. Additionally, it induces non-p53-induced apoptosis and blocks the cell cycle in the late S and G_2_–M phase [97,98,99,100]. Trabectedin has also been found to inhibit cancer cell proliferation, and modulate the TME, acting as an anti-angiogenic and anti-TAM agent [87,101,102,103].

## 6. Passive and Active Targeting

**Figure 1:**
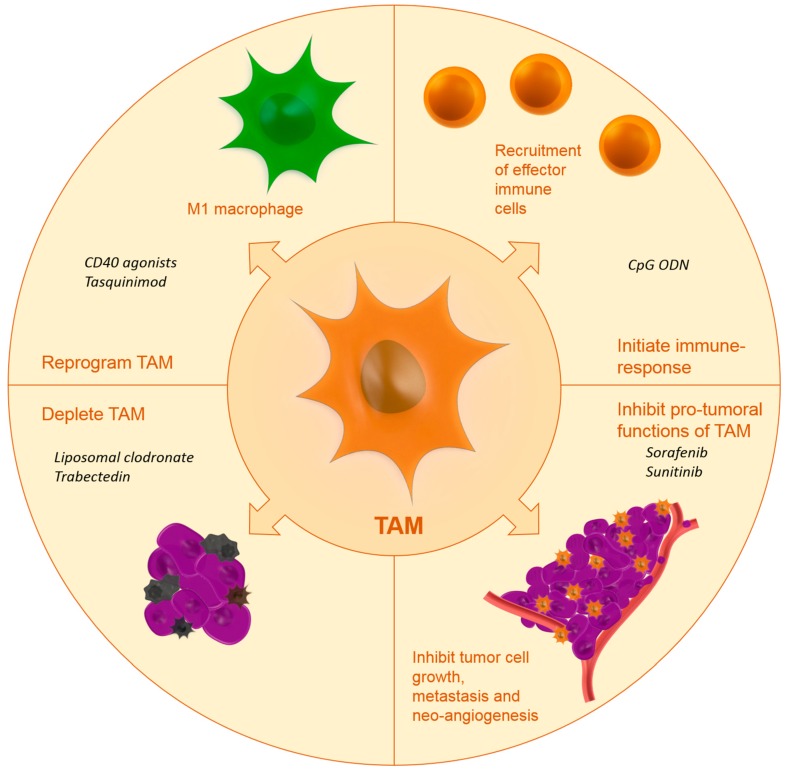
Anti-tumoral effects by targeting TAM using nanocarriers including examples of TAM-modulating therapies (in italics). Abbreviations: CD40: cluster of differentiation 40, CpG ODN: oligodeoxynucleotides containing CpG motifs.

Up until now, few studies have investigated the drug targeting of TAM. Below, we will report on the studies that have investigated TAM targeting using specific carriers or targeting moieties. Figure 1 shows the effects of targeted nanomedicines on TAM.

### 6.1. Passive Targeting

Macrophages are part of the mononuclear phagocyte system (MPS). This system, mostly present in the liver, spleen, and lungs, is responsible for the clearance for foreign matter from the body [104]. As a consequence, nanoparticles that come into contact with macrophages will be rapidly recognized, internalized, and degraded [105,106,107,108]. This intrinsic mechanism of particle uptake by macrophages may be employed to target them. However, reaching certain tissues or specific cell types using passive targeting remains a challenge. Nanoparticle-based therapies, which are being used to target tumor tissues, often rely on the passive enhanced permeation and retention (EPR effect). Due to leaky vasculature and impaired drainage from the tumor site, nanoparticles tend to accumulate in the tumor near the blood vessels [109]. However, penetration of these nanoparticles inside the tumor tissue is often very poor. Moreover, following clinical experience, the EPR effect in humans seems highly variable and limited, possibly due to slow tumor growth, high interstitial fluid pressure, irregular vascular distribution and poor intra-tumoral blood flow [110,111,112]. This issue, together with degree of TAM infiltration, was addressed by imaging of magnetic nanoparticle distribution in tumors and the TME. Magnetic nanoparticles showed clear co-localization with model PLGA–PEG particles, making it possible to identify patients which would be able to benefit from nanoparticle-based therapies [113]. However, even when the EPR effect is limited, macrophages are able to migrate deeper inside tumor tissue, driven by oxygen gradients, towards the hypoxic areas [10,13]. Macrophages may also act as a drug depot, which accumulates drug-loaded nanoparticles and releases them over time [114]. Macrophages may even act as carriers themselves. In this approach, macrophages are loaded with therapeutics ex vivo, after which they will be reintroduced and act as “Trojan Horses” when recruited into the TME. As this strategy has already been reviewed elsewhere, we will not discuss it here [115]. Altogether, due to the EPR effect and the ability of macrophages to phagocytose particles and migrate into tumor areas, nanomedicine may be used to target macrophages and indirectly treat tumor cells. In the sections below, we will discuss a number of nanocarriers used to passively target TAM. A summary of the described studies is displayed in Table 1.

#### 6.1.1. Liposomes

Liposomes are one of the most studied and frequently used carriers in the nanomedicine field. Perhaps the most well-known example of macrophage targeting by liposomes is their depletion by bisphosphonate-loaded liposomes, as mentioned in the “Depletion of TAM” (Section 5.3). Other examples of liposome-based macrophage targeting are described in the section on Active Targeting (Section 6.2). Indeed, due to their composition, enabling encapsulation of hydrophobic, as well as hydrophilic compounds, liposomes have been shown to be excellent carriers for a multitude of different active ingredients, including DNA and siRNA [116]. A nice example of the versatility of liposomes is a study in which pegylated liposomes were loaded with the anti-cancer drug doxorubicin and the bisphosphonate alendronate [117]. Compared to encapsulated doxorubicin, the combination led to superior inhibition of tumor growth in lung and breast cancer mouse models. The authors attribute this enhanced effect to the possible TAM reprogramming by alendronate and heightened immune response due to increased expression of phosphoantigens in tumor cells after exposure to aminobisphosphonates [117]. Another notable example of liposomes as a carrier for the delivery of agents to the TME is the delivery of simvastatin. At high doses, statins have been shown to exert anti-tumor effects [118]. In this study, in order to increase the concentration of simvastatin at the active site, a liposomal delivery system containing simvastatin was used, which was found to inhibit tumor growth, possibly due to the reduction in oxidative stress caused by macrophages, via the inhibition of intra-tumoral HIF-1α [119].

#### 6.1.2. Polymeric Nanoparticles

Polymeric nanoparticles are polymer-based nanoparticles that may take different forms: solid capsules/particles (polymeric nanoparticles), highly branched structures (dendrimers), or amphiphilic assemblies (micelles). Advantages of using polymers are the ability to highly tune nanoparticle properties as well as a high drug-loading capacity [120]. Poly(lactic-*co*-glycolic acid) (PLGA) is a biodegradable copolymer that has been approved for the delivery of therapeutics via sustained release devices and microparticle formulations [121]. Furthermore, next to a wide range of other applications, nanoparticle formulations containing PLGA as a carrier are now being investigated as a delivery mechanism to target TAM. In a recent attempt to reach glioma cells in the brain, rabies virus glycoproteins (RVG), specifically designed to cross the blood brain barrier (BBB), were conjugated to mixed-lipid covered PLGA cores. Although no specific targeting ligand is present on these particles, using in vitro cultured bone marrow derived macrophages the authors were able to show macrophage targeting, which did not occur with neural cells. In vivo results show particle brain accumulation, but specific TAM targeting needs to be confirmed [122].

In a different approach, nanoparticles were prepared using acetylated carboxymethylcellulose. Docetaxel and PEG were coupled via ester linkage (Cellax-DTX). The resulting 120-nm particles were used in different animal models for pancreatic cancer, where specific accumulation in the tumor stroma (specifically fibroblasts and macrophages) was observed, resulting in stromal depletion. However, macrophage populations were able to recover after 2–3 weeks, suggesting that depletion was based on the neutropenic effects of docetaxel rather than macrophage targeting [123].

Linear cyclodextrin polymers have been employed in the preparation of self-assembling nanoparticles. One of the best examples, currently under investigation in clinical trials, is CRLX101. This cyclodextrin-based polymer conjugate is loaded with camptothecin for the treatment of multiple tumor types [124]. Using particles similar to this platform, other applications of cyclodextrin-based polymer–drug conjugates have been investigated as well. One such example is the targeting of intracranial glioma tissues using rhodamine-labeled nanoparticles. Particles that were administered i.v. were predominantly found at the edges of gliomas, internalized by macrophages and microglia. By injecting directly into the tumors, migration of nanoparticle-positive cells was observed into circulation and distant tumor sites, indicating the ability of macrophages and microglia to carry nanoparticles from the injection site towards other affected areas [125].

In an attempt to cross the BBB for the treatment of glioblastoma, hydroxyl-functionalized, generation-4 poly(amidoamine) (PAMAM) dendrimer nanoparticles were intravenously administered to gliosarcoma-bearing rats. Investigators found homogenous distribution of the dendrimers throughout the tumor within 15 minutes after administration, after which retention in microglia and macrophages was observed. The authors propose that dendrimers hold great promise for the delivery of immunomodulatory drugs to TAM across the BBB [126].

#### 6.1.3. Iron Oxide Nanoparticles

Ferumoxytol is an ultrasmall superparamagnetic iron oxide (USPIO) nanoparticle that has been approved for the treatment of anemia due to chronic renal failure. More recently, it has been investigated as a contrast agent in magnetic resonance imaging (MRI). Particles are cleared from the system by the reticuloendothelial system (RES) and macrophages. Due to the uptake of these particles by macrophages, iron oxide nanoparticles have been the subject of investigation in macrophage targeting and imaging [127]. Apart from their favorable imaging properties, plain USPIO have been found to inhibit tumor growth by inducing pro-inflammatory macrophage polarization. In a recent study by Zanganeh et al., in vitro incubation of femuroxytol with co-cultures of macrophages and tumor cells induced tumor cell apoptosis and enhanced macrophage M1 polarization. In vivo, co-implantation of MMTV–PyMT tumor cells with ferumoxytol led to significant inhibition of tumor growth. Analysis of TAM showed an increased presence of pro-inflammatory M1 macrophages in tumor tissues. Moreover, pre-treatment with iron particles prevented liver metastasis after i.v. tumor cell challenge [128]. Considering the available data, USPIO nanoparticles themselves may prove to be promising, not only for imaging, but also for the treatment of TAM.

#### 6.1.4. Biological Carriers

Next to synthetic carriers, biological carriers, mainly due to their favorable immunological functions, may be employed in the targeting and treatment of TAM. Red blood cells (RBCs) have been used as carriers and loaded with various active agents. In one of the earliest studies, prolonged survival was observed after treatment with methotrexate-loaded RBCs in a mouse model of hepatoma ascites tumors [129]. Several other studies have demonstrated the successful loading of bisphosphonates into RBCs for the depletion of macrophages [130,131]. However, these therapies were not tested in disease models. Loaded RBCs in the targeting and treatment of TAM therefore remains to be investigated. High-density lipoprotein (HDL) is another class of biological particles, used in carriers, which, in contrast to RBCs has been shown to be specifically taken up by macrophages, mainly in models for atherosclerosis [132,133]. More recently, these particles, by radioactive labeling, have been used for the imaging of TAM [134]; however, although targeting has been achieved, treatment of TAM using HDL-based carriers has not been attempted yet.

#### 6.1.5. Viral Particles

In line with non-synthetic carriers, a striking article on the use of plant virus particles, specifically interacting with M2 macrophage populations, was published. In this paper, cowpea mosaic virus (CPMV), which is known to interact with vimentin on tumor cells, was prepared as a nanoparticle and the authors demonstrated enhanced uptake by M2-differentiated macrophages [135]. In subsequent studies, however, distinct differences in uptake between M1 and M2 cells were less pronounced, possibly due to different experimental conditions [136].

#### 6.1.6. Carbon Nanotubes

Over the years, carbon nanotubes (CNTs) have received a lot of attention for their use as a biological vector. Moreover, they have been shown to be able to penetrate cells, making them interesting carriers for cell specific targeting [137]. In one of the earliest studies using CNTs to treat brain tumors, after intra-tumoral injection, particles were found to specifically accumulate in TAM, associated with gliomas in mice. Furthermore, no significant toxicities were observed, accompanied by minor changes in tumor cytokine levels [138]. In a study investigating immunotherapy, CpG deoxyoligonucleotides (CpGs) were conjugated to CNTs and injected intratumorally in glioma-bearing mice. Conjugated CpGs were found to be more effective than free CpGs, showing enhanced uptake in TAM. Not only were intracranial gliomas eradicated, but treated mice proved to be immune to subsequent intracranial or systemic tumor challenges, suggesting an induction in systemic anti-tumor immunity [139]. In subsequent studies investigating the effect of CpG-CNT-conjugates on the brain metastasis of primary melanomas, investigators found only a modest inhibition of intracranial melanoma tumors when treating the primary subcutaneous tumor. Intracranial treatment, however, halted both brain and subcutaneous tumor growth. This effect was accompanied by a strong immune response, with increased effector cell infiltration and cytotoxicity. Further investigation into this altered response showed an increase in retention of particles and an increase in infiltration of TLR-9 positive microglia. In contrast, treated subcutaneous tumors showed abundant myeloid-derived suppressor cells. The authors concluded that intracranial delivery of CpG-CNT conjugates is more effective in stimulating immune responses that affect both the brain and subcutaneous melanomas [140].

#### 6.1.7. Albumin Nanoparticles

In addition to other biological carriers, proteins may function as carriers for therapeutics as well. The advantages of using proteins as carrier molecules are their biodegradability (when derived from natural proteins) and their amphiphilic nature, which allows them to interact with drug molecules but also makes them soluble in aqueous environments [141]. The FDA-approved nanoparticle-albumin-bound paclitaxel (nab-paclitaxel, Abraxane) is a good example of this. It is registered for the treatment of advanced breast cancer, advanced non-small cell lung cancer, and advanced pancreatic cancer. Treatment using this conjugate, compared to the free drug, led to a decrease in side effects, increased tumor cell toxicity, and higher overall response rates [142]. Originally, these improved effects were attributed to the higher intra-tumoral concentrations of paclitaxel, due to the binding of albumin to endothelial 60-kDa glycoprotein receptor (gp60), facilitating vascular transcytosis and the binding of albumin to the tumor cell surface receptor secreted protein, acidic and rich in cysteine (SPARC) [143,144,145]. Most recently, an additional mechanism for improved effectiveness was discovered by Cullis et al. [146]. They found that nab-paclitaxel was internalized by macrophages via macropinocytosis. This led to immunostimulatory cytokine and inducible nitric oxide (iNOS) expression. In vivo, nab-paclitaxel was internalized by TAM, leading to increased MHCII^+^ CD80^+^ CD86^+^ M1 macrophage populations. The authors conclude that albumin nanoparticles may be used in the delivery of macrophage-activating agents in the treatment of cancer types with high amounts of M2 macrophage infiltration [146].

#### 6.1.8. Silica Nanoparticles

Although nab-paclitaxel particles have shown potent anti-tumor effects on their own, recently these particles were incorporated into multistage nanovectors (MSVs), consisting of mesoporous silicon nanoparticles, loaded with nab-paclitaxel [147]. By loading nab-paclitaxel into mesoporous silicon nanoparticles, the authors propose a redirection of particles to the liver. In liver metastasis, originating from breast or lung cell lines, authors found decreased liver weights and increased survival of animals treated with loaded MSVs, compared to nab-paclitaxel alone. In subsequent in vitro experiments, macrophages incubated with loaded MSVs were found to form a paclitaxel depot, which was slowly released from the macrophages. The authors conclude that this approach increases therapeutic efficacy without increasing the chance of developing systemic side effects [147]. Since loading into mesoporous silicon nanoparticles dramatically increases the size of the particles, the effect on splenic uptake would be interesting to investigate as well. Indeed, nanoparticle characteristics greatly influence their uptake by different subtypes of macrophages [148]. In a recent study, we investigated the uptake of silica nanoparticles by differentiated macrophages. We found that, with increasing size, the presence of serum proteins greatly influenced the uptake of particles, which was most pronounced in M2 (TAM-like) macrophages. This study indicates that targeting may be achieved by tuning nanoparticle properties, specifically size, in such a way that they are preferentially taken up by specific macrophage populations [149].

### 6.2. Active Targeting

**Figure 2:**
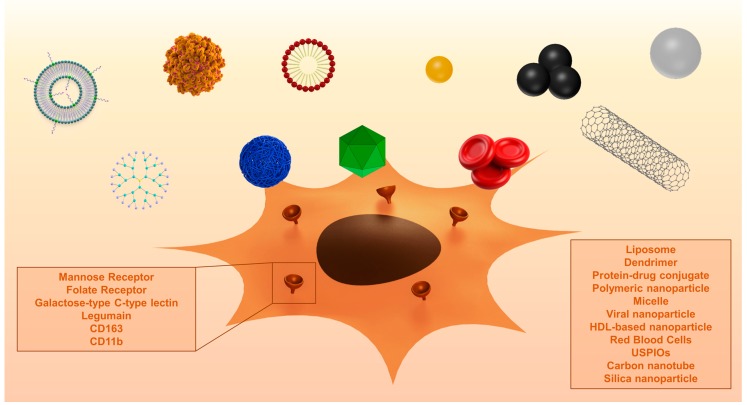
Graphical presentation of surface proteins and different nanoparticles used in TAM targeting. Names of nanoparticles are displayed in their order of appearance, left to right. Abbreviations: HDL: High density lipoproteins, CD: cluster of differentiation, USPIO: ultrasmall superparamagnetic iron oxides.

In recent years, various nanocarriers have been developed. Many of these particles end up in macrophages via endocytosis. Specific targeting of TAM without affecting other macrophage populations is a field of ongoing research. TAMs overexpress different surface receptors, which are exploited for the targeted therapy against these macrophages. Different surface receptors and other proteins that are overexpressed by TAM are discussed below. A summary of the described studies is displayed in Table 2. Figure 2 shows examples of surface proteins that can be targeted using nanoparticles.

#### 6.2.1. Mannose Receptor

The mannose receptor 1 (MRC1, also called CD206) is a scavenger receptor that is overexpressed on the surface of TAMs. Mannose-modified nanoparticles have been used for macrophage targeting to achieve various goals: boosting of the immune responses, imaging, depletion of macrophages, and anti-tumor therapy.

In early studies, mannose-modified cationic liposomes were used for the targeted delivery of genes to enhance the immune response [150,151,152,153]. Hattori et al. compared the delivery of the model gene for ovalbumin encapsulated in normal liposomes and mannosylated liposomes and showed they could increase the immune response by using mannose as a targeting ligand [154]. In subsequent studies, mannose was used for specific TAM targeting and treatment. Locke et al. investigated the accumulation of mannosylated ^64^Cu-loaded liposomes in a mouse model for pulmonary adenocarcinoma using PET imaging and fluorescence microscopy [155]. They compared mannosylated and non-mannosylated liposomes labeled with fluorescent dyes and showed that the mannosylated liposomes accumulated at the tumor regions, while the normal liposomes were distributed throughout the entire lung tissue. Subsequent confocal microscopy confirmed the uptake of the mannosylated liposomes in macrophages expressing MRC1, confirming that mannosylation is a viable approach for targeting TAMs [155]. Similar to the latter study, the polysaccharide mannan, a ligand for the mannose receptor, has been used in several studies for the delivery of nanoparticles to macrophages [156,157]. In a study by Zhan et al., a glucomannan polysaccharide from *Bletilla striata* was employed to target the mannose receptor [158]. The polysaccharide was coupled to the bisphosphonate alendronate for the depletion of macrophages. They showed that a conjugate of the polysaccharide and alendronate preferentially accumulated in macrophages and induced apoptosis in vitro. Furthermore, in a mouse tumor model they showed that depletion of TAM led to inhibition of angiogenesis, recovered local immune surveillance, and suppression of tumor growth [158]. Mannose has also been used in the targeting of polymeric nanoparticles towards TAM. In the past, PEG-sheddable, acid-sensitive, mannose-modified PLGA carriers have been developed and tested for their macrophage targeting potential [159]. In subsequent studies by different investigators, these particles have been tested for their efficacy in cancer treatment [160,161]. One such example is the delivery of acid-sensitive, sheddable, PEGylated, mannose-modified doxorubicin nanoparticles to TAM. In this study, targeted nanoparticles formulations were more effective in reducing tumor growth than non-targeted formulations. Using the targeted formulation, compared to doxorubicin alone, a reduction in the intra-tumoral CD206^+^ cell populations was observed [161].

In another approach using polymers, elegant tri-block copolymer nanoparticles were developed for the delivery of siRNA. In these particles, the core consists of a hydrophobic pH sensitive block, which triggers endosomal escape and enables cytoplasmatic delivery of siRNA. The second block consists of cationic DMAEMA polymers, which are able to condense anionic oligonucleotides within the particle, thus carrying and protecting the cargo. The third block is the azide-presenting block, which enables the particles to be functionalized with targeting molecules, in this case, mannose. Using this functionalized particle, the authors showed the effective transfection of TAM in vitro and in vivo. In primary mammary tumors, the effective delivery of labeled nucleotides was confirmed. Enhanced delivery of labeled nucleotides by targeted particles was established in ovarian tumors, compared to non-targeted particles. Furthermore, in vivo, no significant toxicity was observed [162]. Having established successful targeting of TAM in several mouse models for cancer, investigators then incorporated siRNA for manipulation of the NF-κB pathway. In vitro, the potent induction of cytotoxicity and immunostimulatory functions of TAM was confirmed. In upcoming studies, the authors plan to investigate the effects of this gene silencing in vivo [163,164].

#### 6.2.2. Folate Receptor

Another surface receptor is folate receptor (FR), a glycoprotein that binds to folic acid with high affinity. Initially FR was found to be overexpressed on cancer types of epithelial origin and was thus used for targeted delivery of therapeutics to tumor cells. However, Turk et al. found that FR was also expressed on activated macrophages. In an experimental mouse model of ovarian cancer, they showed a 10-fold higher uptake of folate-conjugated liposomes in TAMs compared to tumor cells, where endocytosis via FR was responsible for 50% of the total uptake [165]. Additional experiments by other groups have shown the presence of the FR-β subtype on the surface of TAMs [166], and the possibility of targeting TAMs using this receptor [167]. In this study, *Pseudomonas exotoxin A* was coupled to an antibody directed against FR-β and injected into C6 glioma xenografts in nude mice. This treatment led to a significant reduction in tumor growth and depletion of TAM. Using this study, the authors concluded that the depletion of FR-β positive macrophages might be a suitable option for immunotherapy of human glioblastoma [167]. Hattori et al. investigated zoledronic-acid-loaded folate modified liposomes for selective TAM depletion. TAM-mediated uptake was observed; however, due to severe toxicity of both conjugated and unconjugated liposomes, no additional anti-tumoral effect could be detected [168].

Folate has also been used in the targeting of USPIO towards TAM. An example is the imaging of TAM in the PyMT mouse mammary tumor model i.v. injected USPIO enhanced MRI signaling in tumor tissues. Upon further investigation, iron oxide nanoparticles were associated with intra-tumoral TAM. In addition, by decorating USPIO nanoparticles with folate, the authors showed increased macrophage uptake [169].

#### 6.2.3. Cluster of Differentiation 163

CD163 is a member of the scavenger receptor cysteine-rich (SRCR) superfamily. It is expressed in most mature macrophages and plays a role in the resolution of inflammation [170,171]. Moreover, it is involved in homeostasis by binding to hemoglobin–haptoglobin complexes [171]. Gordon et al. reported high expression of this receptor in alternatively activated, i.e., M2-like macrophages [172].

Although it is well established that the CD163 receptor is overexpressed on TAM, there is a lack of studies exploiting this receptor for TAM targeting. However, one study attempted to target monocytes by coupling an anti-CD163 antibody via a PEG linker to long circulating liposomes [173]. In vitro, greater uptake in CD163 transfected cells was observed compared to non-transfected cells. When using doxorubicin as a therapeutic agent, cell viability in peripheral blood mononuclear cells was greatly decreased using the targeted construct. The authors conclude that CD163 targeted stealth liposomes may be used to target macrophages in inflammatory and malignant processes.

#### 6.2.4. Legumain

Legumain is a member of the asparaginyl endopeptidase family. It functions as a stress protein, induced by hypoxia, and is overexpressed in TAM, tumor vascular cells, and tumor cells, but not in normal tissues [174,175]. Using a DNA-based vaccine against legumain as a tool, mice were immunized against legumain, leading to depletion of TAM in tumor tissues of 4T1 breast tumor-bearing mice. Depletion of TAM led to a pronounced reduction of TGF-β, TNF-α, MMP-9, and VEGF, resulting in the suppression of angiogenesis, tumor growth, and metastasis. Moreover, vaccination after i.v. tumor cell challenge led to survival of 75% of injected mice, while 62% of them were found to be completely tumor-free [176]. In a nanoparticle-based approach, the small molecular inhibitor for legumain RR-11a was coupled to liposomes. Loading of these particles with doxorubicin led to complete inhibition of tumor growth [177]. In a follow-up study, the legumain-targeted nanoparticle was used for the treatment of TAM. In order to re-educate TAM, hydrazinocurcumin, a synthetic analogue of curcumin able to inhibit the STAT3 pathway, was encapsulated and delivered to TAM. By changing the M2 phenotype of TAM from IL-10^high^/IL-12^low^/TGF-β^high^ to M1 phenotype IL-10^low^/IL-12^high^/TGF-β^low^, authors were able to show TAM re-education in vitro. In vivo, suppressed tumor growth, metastasis, and angiogenesis were observed [178]. Most recently, using an elegant liposomal modification for the simultaneous targeting of TAM, endothelial cells, and tumor cells, the cyclic RGD peptide (iRGD) was combined with a substrate for legumain, alanine-alanine-asparagine (AAN), yielding nRGD [179]. nRGD could specifically bind to legumain, present on tumor endothelial cells, tumor cells, and TAM. Once cleaved by legumain, the remaining nRGD would then be able to bind to αvβ_3_/β_5_ integrin receptors, where it would be cleaved again. The remaining peptide would then bind to neuropilin-1 on tumor cells. The targeted nanoparticle was loaded with doxorubicin and subsequently used in the treatment of 4T1 tumor-bearing mice. Specific interaction with tumor vasculature and efficient tumor penetration were observed. Furthermore, the TME was modulated by depletion of TAM. Overall, the attachment of nRGD to doxorubicin-loaded liposomes led to excellent anti-tumor efficacy, increasing doxorubicin efficacy and low toxicity [179].

#### 6.2.5. Galactose-Type C-Type Lectins

In a novel approach to target TAM for the specific delivery of oligonucleotides, specifically CpG, anti-IL-10, and anti-IL-10R, Huang et al. have developed an acid-sensitive (PEG-histidine modified alginate) nanoparticle, using galactosylated cationic dextran (gal-C-dextran) to form stable nanoplexes able to incorporate and protect oligonucleotides. TAM express high levels of macrophage galactose-type lectin (MGL), making it a suitable target for galactose [180]. In an allograft hepatoma murine model, authors found accumulation of nucleic acids in TAM after i.v. injection. Treatment using the nanoparticles led to significant reduction in tumor growth. Moreover, in TAM isolated from tumor tissues, pro-tumor functions were suppressed and anti-tumor activities were stimulated [181].

#### 6.2.6. Cluster of differentiation 11bCD11b

In another approach to delivering siRNA to macrophages, a glucan-based carrier was developed. Glucans display pathogen-associated molecular patterns (PAMPS), which are recognized and internalized by macrophages. In previous studies, the authors developed water-soluble variants of glucan: β-(1→3)-(1→4)-glucan (BG34). This glucan was found to be internalized by primary monocytes via the CD11b receptor [182]. In a follow-up study, siRNA against MIF was incorporated into this glucan, resulting in 80–120 nm nanoparticles. An i.v. injection of these nanoparticles led to specific tumor accumulation in 4T1 mammary tumor-bearing mice. FACS analysis of TAM isolated from tumor tissues showed a sustained reduction in MIF expression [183].

#### 6.2.7. Peptides

A different way of selectively targeting TAM is the use of peptides. This new approach for targeting TAMs was investigated by Cieslewicz et al. They identified a murine M2 macrophage specific peptide, called M2pep, using subtractive phage biopanning. They showed that this peptide was able to preferentially bind to M2 macrophages, compared to other leukocytes, and they confirmed the accumulation in TAMs in vivo. By fusing M2pep with a pro-apoptotic peptide, they were able to show delayed mortality and a decrease in M2 TAM population in a murine model for colon carcinoma [184].

## 7. Future Perspectives

Specific targeting of TAM is a very recent field of investigation and many hurdles are still remaining. Even though some research has been done to achieve specific targeting of TAM, the expression of the specific markers on other cell types remains a major obstacle. Most active targeting strategies are based on the targeting to upregulated receptors in TAM, but as a matter of fact macrophages often display intermediate states (in between M1 and M2 phenotypes) and other cell types may display these receptors as well [42,185]. Therefore, targeting specificity remains a key challenge. A lack of specificity may lead to treatment of tissue-specific macrophages, such as red pulp macrophages in the spleen or Kupffer cells in the liver. Effects on other macrophage types or receptor-expressing cells should be investigated as well. Moreover, acute toxicity has been observed when administering liposomal zoledronic acid, most likely caused by increased cytokine production [168,186]. Furthermore, the long-term effects of macrophage depletion have yet to be investigated, as there are indications that macrophage depletion may aggravate liver lesions in liver injury and impair skeletal muscle repair after muscle injury [187,188]. However, targeting macrophages seems to be a more effective strategy than identifying tumor specific antigens, as these may change over the course of tumor development and expression may vary between different groups of patients [189]. Recent advances have shown that the EPR effect is not as pronounced in human cancers as in mouse models. Screening of patients in terms of EPR effect and TAM infiltration may increase the number of responders to nanoparticle-based treatments [113,114]. Combining therapy with diagnostics (theranostics), which has been employed in the evaluation of the effectiveness of the targeting and treatment of TAM, might be another useful approach [190].

Production and characterization of complex nanoformulations in GMP conditions may make the translation to the clinic difficult. Production scale-up from the laboratory to the clinic will become more complicated when the particles are prepared in a multi-step process. Therefore, straightforward particles and targeting ligands would be preferable. As shown in several studies, by simply tuning particle characteristics TAM specificity may be increased [149,191]. Preparing them in a single step may make scaling up easier.

## 8. Conclusions

TAM prove to be an interesting therapeutic target for the inhibition of tumor growth and metastasis. Currently, many lines of research are being investigated for the effective delivery of TAM-modulating therapies. A large number of successful attempts have been reported to target TAM via cell-specific surface receptors either to deplete, re-educate, or initiate anti-tumor immune responses. Moreover, the combination of TAM-targeted therapies with conventional medication, directed against the tumor and metastatic sites, holds great promise for effective cancer therapy in the future. Although there are still some hurdles to be overcome, the many preclinical studies reviewed herein show promising results and warrant the further translation of TAM targeting technologies towards the clinic.

## Figures and Tables

**Table 1:** Examples of studies using nanocarriers to passively target TAM.

Carrier Type	Cargo	Model	Purpose	Ref.
Liposomes	Simvastatin	B16.F10 murine melanoma	Improve efficacy of statins using a tumor targeted delivery system	[119]
	Alendronate and doxorubicin	Multiple murine cancer models	Increase anti-tumor efficacy by co-delivery of alendronate and doxorubicin	[117]
Acetylated CMC	Docetaxel	PAN02 pancreatic cancer xenograft	Targeted depletion of stroma	[123]
Linear cyclodextrin-based nanoparticle	Fluorescent label	Murine GL261 glioma	Macrophage and microglia targeting	[125]
Iron oxide nanoparticle	-	Murine breast cancer using MMTV–PyMT cells	Evaluate intrinsic therapeutic effects of USPIO	[128]
Red blood cells	Bisphosphonates	Normal Swiss, C57BL/6 and Balb/C mice	Macrophage depletion	[130,131]
High-density lipoprotein-based nanoparticle	Radiolabel	Murine 4T1 breast cancer	Imaging of TAM	[134]
Cowpea mosaic virus	Fluorescent label, Photosensitizer	RAW264.7 macrophages and B16F10 tumor cells	TAM and tumor cell targeting	[135,136]
PAMAM dendrimers	Fluorescent dye	Rat 9L gliosarcoma	Cross BBB and achieve homogenous tumor distribution	[126]
Carbon nanotubes	Fluorescent dye	Murine GL261 glioma	Study uptake and toxicity	[138]
	CpGs	Murine GL261 glioma	Evaluate CNT as a delivery vehicle	[139,140]
Albumin-paclitaxel conjugate	Paclitaxel	Murine KPC pancreatic ductal adenocarcinoma	Investigate new mechanism for Abraxane effectiveness	[146]
Mesoporous silicon particles loaded with albumin–paclitaxel conjugate	Paclitaxel	Murine 4T1 breast cancer and murine 3LL lung cancer	Redirect nab-paclitaxel to liver metastasis	[147]

Abbreviations: BBB: blood brain barrier, CMC: carboxymethylcellulose, USPIO: ultrasmall superparamagnetic iron oxide, PAMAM: poly(amidoamine), CNT: carbon nanotubes, CpGs: oligodeoxynucleotides containing CpG motifs.

**Table 2:** Examples of studies using nanocarriers to actively target TAM.

Ligand/Target	Carrier	Cargo	Model	Purpose	Ref.
AAN/Legumain	Liposomes	Doxorubicin	Murine 4T1 breast cancer	TAM depletion	[179]
CD163 antibody/CD163	Liposome	Fluorescent label, Doxorubicin	In vitro HEK293, CHO K1 cells and PBMC’s	TAM depletion	[173]
Folate receptor	USPIO	-	MMTV–PyMT murine mammary carcinoma model	Imaging	[169]
	Liposome	Radio- and fluorescent label	Murine IGROV ovarian cancer	Imaging	[165]
		Fluorescent label, zoledronic acid	Several tumor types	TAM depletion	[168]
	FR antibody	Pseudomonas exotoxin A	Human and rat C6 glioma xenografts in mice	TAM depletion	[167]
Galactose/MGL	Alginate-based nanoparticles	CpG, anti-IL-10 and anti-IL-10R oligonucleotides	Hepa 1–6 murine hepatoma	Inhibit pro-tumoral functions and reprogram TAM, initiate immune response	[181]
M2pep/M2 macrophages	Peptide-based nanoparticle	Fluorescent label, pro-apoptotic peptide	Colon carcinoma	Imaging	[184]
Mannose/MR	PLGA	Doxorubicin	Murine M-Wnt triple-negative mammary tumors	TAM depletion	[161]
	BMA-PAA-DMAEMA micelles	siRNA	Multiple tumor types	Reprogramming of TAM	[162,163,164]
	Liposomes	Radiolabel, fluorescent dye	Urethane-FVB pulmonary adenocarcinoma	Imaging	[155]
Polysaccharide from *Bletilla striata*/MR	Polysaccharide from *Bletilla striata*–drug conjugate	Alendronate	Murine S180 sarcoma	TAM depletion	[158]
Rabies virus glycoprotein	PLGA core with mixed lipid coating	Paclitaxel	U87 glioma xenograft	TAM depletion	[122]
RR-11a/Legumain	Liposomes	Hydrazinocurcumin	Murine 4T1 breast cancer	Reprogramming of TAM	[178]
β-(1→3)-(1→4)-glucan/CD11b	Glucan-based nanoparticle	MIF siRNA	Murine 4T1 breast cancer	Inhibit recruitment and reprogramming TAM	[182,183]

Abbreviations: AAN: alanine-alanine-asparagine, PLGA: poly(lactic-*co*-glycolic acid), MR: mannose receptor, BBB: blood brain barrier, BMA: butyl methacrylate, PAA: 2-propylacrylic acid, DMAEMA: 2-(dimethylamino)ethyl methacrylate, CMC: carboxymethylcellulose, FR: folate receptor, USPIO: ultrasmall superparamagnetic iron oxide, CNT: carbon nanotubes, MHC class I: major histocompatibility complex class I, PBMC: peripheral blood mononuclear cell, MGL: macrophage galactose-type C-type lectin, MIF: macrophage migration inhibitory factor, CD11b: cluster of differentiation 11b.

## References

[1] Balkwill F., Mantovani A. (2001). Inflammation and cancer: Back to Virchow?. Lancet.

[2] Brigati C., Noonan D.M., Albini A., Benelli R. (2002). Tumors and inflammatory infiltrates: Friends or foes?. Clin. Exp. Metastasis.

[3] Liotta L.A., Kohn E.C. (2001). The microenvironment of the tumour-host interface. Nature.

[4] Dvorak H.F. (1986). Tumors: Wounds that do not heal. Similarities between tumor stroma generation and wound healing. N. Engl. J. Med..

[5] Hagerling C., Casbon A.J., Werb Z. (2015). Balancing the innate immune system in tumor development. Trends Cell Biol..

[6] Elinav E., Nowarski R., Thaiss C.A., Hu B., Jin C., Flavell R.A. (2013). Inflammation-induced cancer: Crosstalk between tumours, immune cells and microorganisms. Nat. Rev. Cancer.

[7] Mantovani A., Allavena P., Sica A., Balkwill F. (2008). Cancer-related inflammation. Nature.

[8] Koehne C.H., Dubois R.N. (2004). COX-2 inhibition and colorectal cancer. Semin. Oncol..

[9] Flossmann E., Rothwell P.M. (2007). Effect of aspirin on long-term risk of colorectal cancer: Consistent evidence from randomised and observational studies. Lancet.

[10] Sica A., Allavena P., Mantovani A. (2008). Cancer related inflammation: The macrophage connection. Cancer Lett..

[11] Pollard J.W. (2004). Tumour-educated macrophages promote tumour progression and metastasis. Nat. Rev. Cancer.

[12] Allavena P., Mantovani A. (2012). Immunology in the clinic review series; focus on cancer: Tumour-associated macrophages: Undisputed stars of the inflammatory tumour microenvironment. Clin. Exp. Immunol..

[13] Sica A., Schioppa T., Mantovani A., Allavena P. (2006). Tumour-associated macrophages are a distinct M2 polarised population promoting tumour progression: Potential targets of anti-cancer therapy. Eur. J. Cancer.

[14] Shoenfeld Y., Tal A., Berliner S., Pinkhas J. (1986). Leukocytosis in non hematological malignancies—A possible tumor-associated marker. J. Cancer Res. Clin. Oncol..

[15] Fridlender Z.G., Albelda S.M. (2012). Tumor-associated neutrophils: Friend or foe?. Carcinogenesis.

[16] Pillay J., Tak T., Kamp V. M., Koenderman L. (2013). Immune suppression by neutrophils and granulocytic myeloid-derived suppressor cells: Similarities and differences. Cell. Mol. Life Sci..

[17] Dumitru C.A., Lang S., Brandau S. (2013). Modulation of neutrophil granulocytes in the tumor microenvironment: Mechanisms and consequences for tumor progression. Semin. Cancer Biol..

[18] Brandau S., Moses K., Lang S. (2013). The kinship of neutrophils and granulocytic myeloid-derived suppressor cells in cancer: Cousins, siblings or twins?. Semin. Cancer Biol..

[19] Okabe Y., Medzhitov R. (2014). Tissue-specific signals control reversible program of localization and functional polarization of macrophages. Cell.

[20] Locati M., Mantovani A., Sica A. (2013). Macrophage activation and polarization as an adaptive component of innate immunity. Adv. Immunol..

[21] Epelman S., Lavine K.J., Randolph G.J. (2014). Origin and functions of tissue macrophages. Immunity.

[22] Sica A., Erreni M., Allavena P., Porta C. (2015). Macrophage polarization in pathology. Cell. Mol. Life Sci..

[23] Martinez F.O., Gordon S. (2014). The M1 and M2 paradigm of macrophage activation: Time for reassessment. F1000Prime Rep..

[24] Nau G.J., Richmond J.F., Schlesinger A., Jennings E.G., Lander E.S., Young R.A. (2002). Human macrophage activation programs induced by bacterial pathogens. Proc. Natl. Acad. Sci. USA.

[25] Mantovani A., Sica A., Sozzani S., Allavena P., Vecchi A., Locati M. (2004). The chemokine system in diverse forms of macrophage activation and polarization. Trends Immunol..

[26] Heusinkveld M., van der Burg S.H. (2011). Identification and manipulation of tumor associated macrophages in human cancers. J. Transl. Med..

[27] Hao N.B., Lu M.H., Fan Y.H., Cao Y.L., Zhang Z.R., Yang S.M. (2012). Macrophages in tumor microenvironments and the progression of tumors. Clin. Dev. Immunol..

[28] Biswas S.K., Mantovani A. (2010). Macrophage plasticity and interaction with lymphocyte subsets: Cancer as a paradigm. Nat. Immunol..

[29] Mills C.D. (2012). M1 and M2 macrophages: Oracles of health and disease. Crit. Rev. Immunol..

[30] Mills C.D., Shearer J., Evans R., Caldwell M.D. (1992). Macrophage arginine metabolism and the inhibition or stimulation of cancer. J. Immunol..

[31] Heusinkveld M., de Vos van Steenwijk P.J., Goedemans R., Ramwadhdoebe T.H., Gorter A., Welters M.J., van Hall T., van der Burg S.H. (2011). M2 macrophages induced by prostaglandin E2 and IL-6 from cervical carcinoma are switched to activated M1 macrophages by CD4+ Th1 cells. J. Immunol..

[32] Fujiwara Y., Komohara Y., Kudo R., Tsurushima K., Ohnishi K., Ikeda T., Takeya M. (2011). Oleanolic acid inhibits macrophage differentiation into the M2 phenotype and glioblastoma cell proliferation by suppressing the activation of STAT3. Oncol. Rep..

[33] Oishi K., Sakaguchi T., Baba S., Suzuki S., Konno H. (2015). Macrophage density and macrophage colony-stimulating factor expression predict the postoperative prognosis in patients with intrahepatic cholangiocarcinoma. Surg. Today.

[34] Ding T., Xu J., Wang F., Shi M., Zhang Y., Li S.P., Zheng L. (2009). High tumor-infiltrating macrophage density predicts poor prognosis in patients with primary hepatocellular carcinoma after resection. Hum. Pathol..

[35] Mantovani A., Allavena P., Sica A. (2004). Tumour-associated macrophages as a prototypic type II polarised phagocyte population: Role in tumour progression. Eur. J. Cancer.

[36] Colegio O.R., Chu N.Q., Szabo A.L., Chu T., Rhebergen A.M., Jairam V., Cyrus N., Brokowski C.E., Eisenbarth S.C., Phillips G.M. (2014). Functional polarization of tumour-associated macrophages by tumour-derived lactic acid. Nature.

[37] Sica A., Saccani A., Bottazzi B., Polentarutti N., Vecchi A., van Damme J., Mantovani A. (2000). Autocrine production of IL-10 mediates defective IL-12 production and NF-κB activation in tumor-associated macrophages. J. Immunol..

[38] Rebe C., Vegran F., Berger H., Ghiringhelli F. (2013). STAT3 activation: A key factor in tumor immunoescape. JAKSTAT.

[39] Zhang Y., Choksi S., Chen K., Pobezinskaya Y., Linnoila I., Liu Z.G. (2013). ROS play a critical role in the differentiation of alternatively activated macrophages and the occurrence of tumor-associated macrophages. Cell Res..

[40] Yuan Z.Y., Luo R.Z., Peng R.J., Wang S.S., Xue C. (2014). High infiltration of tumor-associated macrophages in triple-negative breast cancer is associated with a higher risk of distant metastasis. Onco Targets Ther..

[41] Kubler K., Ayub T.H., Weber S.K., Zivanovic O., Abramian A., Keyver-Paik M.D., Mallmann M.R., Kaiser C., Serce N.B., Kuhn W. (2014). Prognostic significance of tumor-associated macrophages in endometrial adenocarcinoma. Gynecol. Oncol..

[42] Biswas S.K., Allavena P., Mantovani A. (2013). Tumor-associated macrophages: Functional diversity, clinical significance, and open questions. Semin. Immunopathol..

[43] Qian B.Z., Pollard J.W. (2010). Macrophage diversity enhances tumor progression and metastasis. Cell.

[44] Lin E.Y., Nguyen A.V., Russell R.G., Pollard J.W. (2001). Colony-stimulating factor 1 promotes progression of mammary tumors to malignancy. J. Exp. Med..

[45] Wyckoff J., Wang W., Lin E.Y., Wang Y., Pixley F., Stanley E.R., Graf T., Pollard J.W., Segall J., Condeelis J. (2004). A paracrine loop between tumor cells and macrophages is required for tumor cell migration in mammary tumors. Cancer Res..

[46] Wyckoff J.B., Wang Y., Lin E.Y., Li J.F., Goswami S., Stanley E.R., Segall J.E., Pollard J.W., Condeelis J. (2007). Direct visualization of macrophage-assisted tumor cell intravasation in mammary tumors. Cancer Res..

[47] Hagemann T., Robinson S.C., Schulz M., Trumper L., Balkwill F.R., Binder C. (2004). Enhanced invasiveness of breast cancer cell lines upon co-cultivation with macrophages is due to TNF-α dependent up-regulation of matrix metalloproteases. Carcinogenesis.

[48] Ingman W.V., Wyckoff J., Gouon-Evans V., Condeelis J., Pollard J.W. (2006). Macrophages promote collagen fibrillogenesis around terminal end buds of the developing mammary gland. Dev. Dyn..

[49] Condeelis J., Segall J.E. (2003). Intravital imaging of cell movement in tumours. Nat. Rev. Cancer.

[50] Du R., Lu K.V., Petritsch C., Liu P., Ganss R., Passegue E., Song H., Vandenberg S., Johnson R.S., Werb Z. (2008). HIF1α induces the recruitment of bone marrow-derived vascular modulatory cells to regulate tumor angiogenesis and invasion. Cancer Cell.

[51] Casazza A., Laoui D., Wenes M., Rizzolio S., Bassani N., Mambretti M., Deschoemaeker S., van Ginderachter J.A., Tamagnone L., Mazzone M. (2013). Impeding macrophage entry into hypoxic tumor areas by Sema3A/Nrp1 signaling blockade inhibits angiogenesis and restores antitumor immunity. Cancer Cell.

[52] Guruvayoorappan C. (2008). Tumor versus tumor-associated macrophages: How hot is the link?. Integr. Cancer Ther..

[53] Mantovani A., Savino B., Locati M., Zammataro L., Allavena P., Bonecchi R. (2010). The chemokine system in cancer biology and therapy. Cytokine Growth Factor Rev..

[54] Lazennec G., Richmond A. (2010). Chemokines and chemokine receptors: New insights into cancer-related inflammation. Trends Mol. Med..

[55] Gazzaniga S., Bravo A.I., Guglielmotti A., van Rooijen N., Maschi F., Vecchi A., Mantovani A., Mordoh J., Wainstok R. (2007). Targeting tumor-associated macrophages and inhibition of MCP-1 reduce angiogenesis and tumor growth in a human melanoma xenograft. J. Investig. Dermatol..

[56] Pienta K.J., Machiels J.P., Schrijvers D., Alekseev B., Shkolnik M., Crabb S.J., Li S., Seetharam S., Puchalski T.A., Takimoto C. (2013). Phase 2 study of carlumab (CNTO 888), a human monoclonal antibody against CC-chemokine ligand 2 (CCL2), in metastatic castration-resistant prostate cancer. Investig. New Drugs.

[57] Sandhu S.K., Papadopoulos K., Fong P.C., Patnaik A., Messiou C., Olmos D., Wang G., Tromp B.J., Puchalski T.A., Balkwill F. (2013). A first-in-human, first-in-class, phase I study of carlumab (CNTO 888), a human monoclonal antibody against CC-chemokine ligand 2 in patients with solid tumors. Cancer Chemother. Pharmacol..

[58] ClinicalTrials.gov, N.L.o.H. MLN1202 in Treating Patients with Bone Metastases. https://clinicaltrials.gov/ct2/show/NCT01015560.

[59] Ahn G.O., Tseng D., Liao C.H., Dorie M.J., Czechowicz A., Brown J.M. (2010). Inhibition of Mac-1 (CD11b/CD18) enhances tumor response to radiation by reducing myeloid cell recruitment. Proc. Natl. Acad. Sci. USA.

[60] Arnaout M.A. (1990). Structure and function of the leukocyte adhesion molecules CD11/CD18. Blood.

[61] Prada C.E., Jousma E., Rizvi T.A., Wu J., Dunn R.S., Mayes D.A., Cancelas J.A., Dombi E., Kim M.O., West B.L. (2013). Neurofibroma-associated macrophages play roles in tumor growth and response to pharmacological inhibition. Acta Neuropathol..

[62] Strachan D.C., Ruffell B., Oei Y., Bissell M.J., Coussens L.M., Pryer N., Daniel D. (2013). CSF1R inhibition delays cervical and mammary tumor growth in murine models by attenuating the turnover of tumor-associated macrophages and enhancing infiltration by CD8 T cells. Oncoimmunology.

[63] Weizman N., Krelin Y., Shabtay-Orbach A., Amit M., Binenbaum Y., Wong R.J., Gil Z. (2014). Macrophages mediate gemcitabine resistance of pancreatic adenocarcinoma by upregulating cytidine deaminase. Oncogene.

[64] Pyonteck S.M., Akkari L., Schuhmacher A.J., Bowman R.L., Sevenich L., Quail D.F., Olson O.C., Quick M.L., Huse J.T., Teijeiro V. (2013). CSF-1R inhibition alters macrophage polarization and blocks glioma progression. Nat. Med..

[65] Sluijter M., van der Sluis T.C., van der Velden P.A., Versluis M., West B.L., van der Burg S.H., van Hall T. (2014). Inhibition of CSF-1R supports T-cell mediated melanoma therapy. PLoS ONE.

[66] Kim T.S., Cavnar M.J., Cohen N.A., Sorenson E.C., Greer J.B., Seifert A.M., Crawley M.H., Green B.L., Popow R., Pillarsetty N. (2014). Increased KIT inhibition enhances therapeutic efficacy in gastrointestinal stromal tumor. Clin. Cancer Res..

[67] Patwardhan P.P., Surriga O., Beckman M.J., de Stanchina E., Dematteo R.P., Tap W.D., Schwartz G.K. (2014). Sustained inhibition of receptor tyrosine kinases and macrophage depletion by PLX3397 and rapamycin as a potential new approach for the treatment of MPNSTs. Clin. Cancer Res..

[68] Ries C.H., Cannarile M.A., Hoves S., Benz J., Wartha K., Runza V., Rey-Giraud F., Pradel L.P., Feuerhake F., Klaman I. (2014). Targeting tumor-associated macrophages with anti-CSF-1R antibody reveals a strategy for cancer therapy. Cancer Cell.

[69] Roche Investor Update. http://www.roche.com/investors/updates/inv-update-2014-06-01.htm.

[70] Duluc D., Corvaisier M., Blanchard S., Catala L., Descamps P., Gamelin E., Ponsoda S., Delneste Y., Hebbar M., Jeannin P. (2009). Interferon-gamma reverses the immunosuppressive and protumoral properties and prevents the generation of human tumor-associated macrophages. Int. J. Cancer.

[71] Tang X., Mo C., Wang Y., Wei D., Xiao H. (2013). Anti-tumour strategies aiming to target tumour-associated macrophages. Immunology.

[72] Beatty G.L., Chiorean E.G., Fishman M.P., Saboury B., Teitelbaum U.R., Sun W., Huhn R.D., Song W., Li D., Sharp L.L. (2011). CD40 agonists alter tumor stroma and show efficacy against pancreatic carcinoma in mice and humans. Science.

[73] Buhtoiarov I.N., Lum H., Berke G., Paulnock D.M., Sondel P.M., Rakhmilevich A.L. (2005). CD40 ligation activates murine macrophages via an IFN-γ-dependent mechanism resulting in tumor cell destruction in vitro. J. Immunol..

[74] Buhtoiarov I.N., Lum H.D., Berke G., Sondel P.M., Rakhmilevich A.L. (2006). Synergistic activation of macrophages via CD40 and TLR9 results in T cell independent antitumor effects. J. Immunol..

[75] Jensen J.L., Rakhmilevich A., Heninger E., Broman A.T., Hope C., Phan F., Miyamoto S., Maroulakou I., Callander N., Hematti P. (2015). Tumoricidal effects of macrophage-activating immunotherapy in a murine model of relapsed/refractory multiple myeloma. Cancer Immunol. Res..

[76] Mantovani A., Marchesi F., Malesci A., Laghi L., Allavena P. (2017). Tumour-associated macrophages as treatment targets in oncology. Nat. Rev. Clin. Oncol..

[77] Olsson A., Nakhle J., Sundstedt A., Plas P., Bauchet A.L., Pierron V., Bruetschy L., Deronic A., Torngren M., Liberg D. (2015). Tasquinimod triggers an early change in the polarization of tumor associated macrophages in the tumor microenvironment. J. Immunother. Cancer.

[78] Shen L., Sundstedt A., Ciesielski M., Miles K.M., Celander M., Adelaiye R., Orillion A., Ciamporcero E., Ramakrishnan S., Ellis L. (2015). Tasquinimod modulates suppressive myeloid cells and enhances cancer immunotherapies in murine models. Cancer Immunol. Res..

[79] White E.S., Flaherty K.R., Carskadon S., Brant A., Iannettoni M.D., Yee J., Orringer M.B., Arenberg D.A. (2003). Macrophage migration inhibitory factor and CXC chemokine expression in non-small cell lung cancer: Role in angiogenesis and prognosis. Clin. Cancer Res..

[80] White E.S., Strom S.R., Wys N.L., Arenberg D.A. (2001). Non-small cell lung cancer cells induce monocytes to increase expression of angiogenic activity. J. Immunol..

[81] Yaddanapudi K., Putty K., Rendon B.E., Lamont G.J., Faughn J.D., Satoskar A., Lasnik A., Eaton J.W., Mitchell R.A. (2013). Control of tumor-associated macrophage alternative activation by macrophage migration inhibitory factor. J. Immunol..

[82] Cheng F., Wang H.W., Cuenca A., Huang M., Ghansah T., Brayer J., Kerr W.G., Takeda K., Akira S., Schoenberger S.P. (2003). A critical role for Stat3 signaling in immune tolerance. Immunity.

[83] Xin H., Zhang C., Herrmann A., Du Y., Figlin R., Yu H. (2009). Sunitinib inhibition of Stat3 induces renal cell carcinoma tumor cell apoptosis and reduces immunosuppressive cells. Cancer Res..

[84] Edwards J.P., Emens L.A. (2010). The multikinase inhibitor sorafenib reverses the suppression of IL-12 and enhancement of IL-10 by PGE(2) in murine macrophages. Int. Immunopharmacol..

[85] Hebenstreit D., Wirnsberger G., Horejs-Hoeck J., Duschl A. (2006). Signaling mechanisms, interaction partners, and target genes of STAT6. Cytokine Growth Factor Rev..

[86] Binnemars-Postma K., Bansal R., Storm G., Prakash J. (2015). 355 Targeting the STAT6 pathway to inhibit tumor-associated macrophages-induced tumor growth and metastasis in breast cancer. Eur. J. Cancer.

[87] Germano G., Frapolli R., Belgiovine C., Anselmo A., Pesce S., Liguori M., Erba E., Uboldi S., Zucchetti M., Pasqualini F. (2013). Role of macrophage targeting in the antitumor activity of trabectedin. Cancer Cell.

[88] Rogers T.L., Holen I. (2011). Tumour macrophages as potential targets of bisphosphonates. J. Transl. Med..

[89] Fleisch H. (1989). Bisphosphonates: A new class of drugs in diseases of bone and calcium metabolism. Recent Results Cancer Res..

[90] Van Rooijen N., van Nieuwmegen R. (1984). Elimination of phagocytic cells in the spleen after intravenous injection of liposome-encapsulated dichloromethylene diphosphonate. An enzyme-histochemical study. Cell Tissue Res..

[91] Brown H.K., Holen I. (2009). Anti-tumour effects of bisphosphonates—What have we learned from in vivo models?. Curr. Cancer Drug Targets.

[92] Zeisberger S.M., Odermatt B., Marty C., Zehnder-Fjallman A.H., Ballmer-Hofer K., Schwendener R.A. (2006). Clodronate-liposome-mediated depletion of tumour-associated macrophages: A new and highly effective antiangiogenic therapy approach. Br. J. Cancer.

[93] Wu X., Schulte B.C., Zhou Y., Haribhai D., Mackinnon A.C., Plaza J.A., Williams C.B., Hwang S.T. (2014). Depletion of M2-like tumor-associated macrophages delays cutaneous t-cell lymphoma development in vivo. J. Investig. Dermatol..

[94] Zhang W., Zhu X.D., Sun H.C., Xiong Y.Q., Zhuang P.Y., Xu H.X., Kong L.Q., Wang L., Wu W.Z., Tang Z.Y. (2010). Depletion of tumor-associated macrophages enhances the effect of sorafenib in metastatic liver cancer models by antimetastatic and antiangiogenic effects. Clin. Cancer Res..

[95] Junankar S., Shay G., Jurczyluk J., Ali N., Down J., Pocock N., Parker A., Nguyen A., Sun S., Kashemirov B. (2015). Real-time intravital imaging establishes tumor-associated macrophages as the extraskeletal target of bisphosphonate action in cancer. Cancer Discov..

[96] Guan Y., Sakai R., Rinehart K.L., Wang A.H. (1993). Molecular and crystal structures of ecteinascidins: Potent antitumor compounds from the Caribbean tunicate *Ecteinascidia turbinata*. J. Biomol. Struct. Dyn..

[97] Allavena P., Signorelli M., Chieppa M., Erba E., Bianchi G., Marchesi F., Olimpio C.O., Bonardi C., Garbi A., Lissoni A. (2005). Anti-inflammatory properties of the novel antitumor agent yondelis (trabectedin): Inhibition of macrophage differentiation and cytokine production. Cancer Res..

[98] Pommier Y., Kohlhagen G., Bailly C., Waring M., Mazumder A., Kohn K.W. (1996). DNA sequence- and structure-selective alkylation of guanine N2 in the DNA minor groove by ecteinascidin 743, a potent antitumor compound from the Caribbean tunicate *Ecteinascidia turbinata*. Biochemistry.

[99] Zewail-Foote M., Hurley L.H. (1999). Ecteinascidin 743: A minor groove alkylator that bends DNA toward the major groove. J. Med. Chem..

[100] Erba E., Bergamaschi D., Bassano L., Damia G., Ronzoni S., Faircloth G.T., D′Incalci M. (2001). Ecteinascidin-743 (ET-743), a natural marine compound, with a unique mechanism of action. Eur. J. Cancer.

[101] D’Incalci M., Zambelli A. (2016). Trabectedin for the treatment of breast cancer. Expert Opin. Investig. Drugs.

[102] Germano G., Frapolli R., Simone M., Tavecchio M., Erba E., Pesce S., Pasqualini F., Grosso F., Sanfilippo R., Casali P.G. (2010). Antitumor and anti-inflammatory effects of trabectedin on human myxoid liposarcoma cells. Cancer Res..

[103] Atmaca H., Uzunoglu S. (2014). Anti-angiogenic effects of trabectedin (Yondelis; ET-743) on human breast cancer cells. Eur. Cytokine Netw..

[104] Flannagan R.S., Jaumouille V., Grinstein S. (2012). The cell biology of phagocytosis. Annu. Rev. Pathol..

[105] Alexis F., Pridgen E., Molnar L.K., Farokhzad O.C. (2008). Factors affecting the clearance and biodistribution of polymeric nanoparticles. Mol. Pharm..

[106] Haniffa M., Bigley V., Collin M. (2015). Human mononuclear phagocyte system reunited. Semin. Cell Dev. Biol..

[107] Liu T., Choi H., Zhou R., Chen I.W. (2014). Quantitative evaluation of the reticuloendothelial system function with dynamic MRI. PLoS ONE.

[108] Owens D.E., Peppas N.A. (2006). Opsonization, biodistribution, and pharmacokinetics of polymeric nanoparticles. Int. J. Pharm..

[109] Maeda H., Nakamura H., Fang J. (2013). The EPR effect for macromolecular drug delivery to solid tumors: Improvement of tumor uptake, lowering of systemic toxicity, and distinct tumor imaging in vivo. Adv. Drug Deliv. Rev..

[110] Nichols J.W., Bae Y.H. (2014). EPR: Evidence and fallacy. J. Control Release.

[111] Shi J., Kantoff P.W., Wooster R., Farokhzad O.C. (2017). Cancer nanomedicine: Progress, challenges and opportunities. Nat. Rev. Cancer.

[112] Anchordoquy T.J., Barenholz Y., Boraschi D., Chorny M., Decuzzi P., Dobrovolskaia M.A., Farhangrazi Z.S., Farrell D., Gabizon A., Ghandehari H. (2017). Mechanisms and barriers in cancer nanomedicine: Addressing challenges, looking for solutions. ACS Nano.

[113] Miller M.A., Gadde S., Pfirschke C., Engblom C., Sprachman M.M., Kohler R.H., Yang K.S., Laughney A.M., Wojtkiewicz G., Kamaly N. (2015). Predicting therapeutic nanomedicine efficacy using a companion magnetic resonance imaging nanoparticle. Sci. Transl. Med..

[114] Miller M.A., Zheng Y.R., Gadde S., Pfirschke C., Zope H., Engblom C., Kohler R.H., Iwamoto Y., Yang K.S., Askevold B. (2015). Tumour-associated macrophages act as a slow-release reservoir of nano-therapeutic Pt(IV) pro-drug. Nat. Commun..

[115] Si J., Shao S., Shen Y., Wang K. (2016). Macrophages as active nanocarriers for targeted early and adjuvant cancer chemotherapy. Small.

[116] Allen T.M., Cullis P.R. (2013). Liposomal drug delivery systems: From concept to clinical applications. Adv. Drug Deliv. Rev..

[117] Shmeeda H., Amitay Y., Gorin J., Tzemach D., Mak L., Stern S.T., Barenholz Y., Gabizon A. (2016). Coencapsulation of alendronate and doxorubicin in pegylated liposomes: A novel formulation for chemoimmunotherapy of cancer. J. Drug Target.

[118] Qi X.F., Kim D.H., Yoon Y.S., Kim S.K., Cai D.Q., Teng Y.C., Shim K.Y., Lee K.J. (2010). Involvement of oxidative stress in simvastatin-induced apoptosis of murine CT26 colon carcinoma cells. Toxicol. Lett..

[119] Alupei M.C., Licarete E., Patras L., Banciu M. (2015). Liposomal simvastatin inhibits tumor growth via targeting tumor-associated macrophages-mediated oxidative stress. Cancer Lett..

[120] Perez-Herrero E., Fernandez-Medarde A. (2015). Advanced targeted therapies in cancer: Drug nanocarriers, the future of chemotherapy. Eur. J. Pharm. Biopharm..

[121] Peres C., Matos A.I., Conniot J., Sainz V., Zupancic E., Silva J.M., Graca L., Sa Gaspar R., Preat V., Florindo H.F. (2017). Poly(lactic acid)-based particulate systems are promising tools for immune modulation. Acta Biomater..

[122] Zou L., Tao Y., Payne G., Do L., Thomas T., Rodriguez J., Dou H. (2017). Targeted delivery of nano-PTX to the brain tumor-associated macrophages. Oncotarget.

[123] Ernsting M.J., Hoang B., Lohse I., Undzys E., Cao P., Do T., Gill B., Pintilie M., Hedley D., Li S.D. (2015). Targeting of metastasis-promoting tumor-associated fibroblasts and modulation of pancreatic tumor-associated stroma with a carboxymethylcellulose-docetaxel nanoparticle. J. Control Release.

[124] Cerulean Pharma Inc. Platform & Pipeline CRLX101. http://ceruleanrx.com/platform-pipeline/crlx101.php.

[125] Alizadeh D., Zhang L., Hwang J., Schluep T., Badie B. (2010). Tumor-associated macrophages are predominant carriers of cyclodextrin-based nanoparticles into gliomas. Nanomedicine.

[126] Zhang F., Mastorakos P., Mishra M.K., Mangraviti A., Hwang L., Zhou J., Hanes J., Brem H., Olivi A., Tyler B. (2015). Uniform brain tumor distribution and tumor associated macrophage targeting of systemically administered dendrimers. Biomaterials.

[127] Bashir M.R., Bhatti L., Marin D., Nelson R.C. (2015). Emerging applications for ferumoxytol as a contrast agent in MRI. J. Magn. Reson. Imaging.

[128] Zanganeh S., Hutter G., Spitler R., Lenkov O., Mahmoudi M., Shaw A., Pajarinen J.S., Nejadnik H., Goodman S., Moseley M. (2016). Iron oxide nanoparticles inhibit tumour growth by inducing pro-inflammatory macrophage polarization in tumour tissues. Nat. Nanotechnol..

[129] Kruse C.A., Freehauf C.L., Patel K.R., Baldeschwieler J.D. (1987). Mouse erythrocyte carriers osmotically loaded with methotrexate. Biotechnol. Appl. Biochem..

[130] Rossi L., Serafini S., Antonelli A., Pierige F., Carnevali A., Battistelli V., Malatesta M., Balestra E., Calio R., Perno C.F. (2005). Macrophage depletion induced by clodronate-loaded erythrocytes. J. Drug Target.

[131] Sabatino R., Antonelli A., Battistelli S., Schwendener R., Magnani M., Rossi L. (2014). Macrophage depletion by free bisphosphonates and zoledronate-loaded red blood cells. PLoS ONE.

[132] Duivenvoorden R., Tang J., Cormode D.P., Mieszawska A.J., Izquierdo-Garcia D., Ozcan C., Otten M.J., Zaidi N., Lobatto M.E., van Rijs S.M. (2014). A statin-loaded reconstituted high-density lipoprotein nanoparticle inhibits atherosclerotic plaque inflammation. Nat. Commun..

[133] Tang J., Lobatto M.E., Hassing L., van der Staay S., van Rijs S.M., Calcagno C., Braza M.S., Baxter S., Fay F., Sanchez-Gaytan B.L. (2015). Inhibiting macrophage proliferation suppresses atherosclerotic plaque inflammation. Sci. Adv..

[134] Perez-Medina C., Tang J., Abdel-Atti D., Hogstad B., Merad M., Fisher E.A., Fayad Z.A., Lewis J.S., Mulder W.J., Reiner T. (2015). PET imaging of tumor-associated macrophages with ^89^Zr-labeled high-density lipoprotein nanoparticles. J. Nucl. Med..

[135] Agrawal A., Manchester M. (2012). Differential uptake of chemically modified cowpea mosaic virus nanoparticles in macrophage subpopulations present in inflammatory and tumor microenvironments. Biomacromolecules.

[136] Wen A.M., Lee K.L., Cao P., Pangilinan K., Carpenter B.L., Lam P., Veliz F.A., Ghiladi R.A., Advincula R.C., Steinmetz N.F. (2016). Utilizing viral nanoparticle/dendron hybrid conjugates in photodynamic therapy for dual delivery to macrophages and cancer cells. Bioconjug. Chem..

[137] Klumpp C., Kostarelos K., Prato M., Bianco A. (2006). Functionalized carbon nanotubes as emerging nanovectors for the delivery of therapeutics. Biochim. Biophys. Acta.

[138] VanHandel M., Alizadeh D., Zhang L., Kateb B., Bronikowski M., Manohara H., Badie B. (2009). Selective uptake of multi-walled carbon nanotubes by tumor macrophages in a murine glioma model. J. Neuroimmunol..

[139] Zhao D., Alizadeh D., Zhang L., Liu W., Farrukh O., Manuel E., Diamond D.J., Badie B. (2011). Carbon nanotubes enhance CpG uptake and potentiate antiglioma immunity. Clin. Cancer Res..

[140] Fan H., Zhang I., Chen X., Zhang L., Wang H., Da Fonseca A., Manuel E.R., Diamond D.J., Raubitschek A., Badie B. (2012). Intracerebral CpG immunotherapy with carbon nanotubes abrogates growth of subcutaneous melanomas in mice. Clin. Cancer Res..

[141] Lohcharoenkal W., Zhang I., Chen X., Zhang L., Wang H., Da Fonseca A., Manuel E.R., Diamond D.J., Raubitschek A., Badie B. (2014). Protein nanoparticles as drug delivery carriers for cancer therapy. BioMed Res. Int..

[142] Kundranda M.N., Niu J. (2015). Albumin-bound paclitaxel in solid tumors: Clinical development and future directions. Drug Des. Dev. Ther..

[143] Desai N., Trieu V., Yao Z., Louie L., Ci S., Yang A., Tao C., De T., Beals B., Dykes D. (2006). Increased antitumor activity, intratumor paclitaxel concentrations, and endothelial cell transport of cremophor-free, albumin-bound paclitaxel, ABI-007, compared with cremophor-based paclitaxel. Clin. Cancer Res..

[144] Desai N.P., Trieu V., Hwang L.Y., Wu R., Soon-Shiong P., Gradishar W.J. (2008). Improved effectiveness of nanoparticle albumin-bound (nab) paclitaxel versus polysorbate-based docetaxel in multiple xenografts as a function of HER2 and SPARC status. Anticancer Drugs.

[145] Desai N., Trieu V., Damascelli B., Soon-Shiong P. (2009). SPARC expression correlates with tumor response to albumin-bound paclitaxel in head and neck cancer patients. Transl. Oncol..

[146] Cullis J., Siolas D., Avanzi A., Barui S., Maitra A., Bar-Sagi D. (2017). Macropinocytosis of nab-paclitaxel drives macrophage activation in pancreatic cancer. Cancer Immunol. Res..

[147] Tanei T., Leonard F., Liu X., Alexander J.F., Saito Y., Ferrari M., Godin B., Yokoi K. (2016). Redirecting transport of nanoparticle albumin-bound paclitaxel to macrophages enhances therapeutic efficacy against liver metastases. Cancer Res..

[148] Verma A., Stellacci F. (2010). Effect of surface properties on nanoparticle-cell interactions. Small.

[149] Binnemars-Postma K.A., Ten Hoopen H.W., Storm G., Prakash J. (2016). Differential uptake of nanoparticles by human M1 and M2 polarized macrophages: Protein corona as a critical determinant. Nanomedicine.

[150] Perrie Y., Frederik P.M., Gregoriadis G. (2001). Liposome-mediated DNA vaccination: The effect of vesicle composition. Vaccine.

[151] Ishii N., Fukushima J., Kaneko T., Okada E., Tani K., Tanaka S.I., Hamajima K., Xin K.Q., Kawamoto S., Koff W. (1997). Cationic liposomes are a strong adjuvant for a DNA vaccine of human immunodeficiency virus type 1. AIDS Res. Hum. Retroviruses.

[152] Tanaka M., Kaneda Y., Fujii S., Yamano T., Hashimoto K., Huang S.K., Hoon D.S. (2002). Induction of a systemic immune response by a polyvalent melanoma-associated antigen DNA vaccine for prevention and treatment of malignant melanoma. Mol. Ther..

[153] Toda S., Ishii N., Okada E., Kusakabe K.I., Arai H., Hamajima K., Gorai I., Nishioka K., Okuda K. (1997). HIV-1-specific cell-mediated immune responses induced by DNA vaccination were enhanced by mannan-coated liposomes and inhibited by anti-interferon-gamma antibody. Immunology.

[154] Hattori Y., Kawakami S., Suzuki S., Yamashita F., Hashida M. (2004). Enhancement of immune responses by DNA vaccination through targeted gene delivery using mannosylated cationic liposome formulations following intravenous administration in mice. Biochem. Biophys. Res. Commun..

[155] Locke L.W., Mayo M.W., Yoo A.D., Williams M.B., Berr S.S. (2012). PET imaging of tumor associated macrophages using mannose coated ^64^Cu liposomes. Biomaterials.

[156] Kaur A., Jain S., Tiwary A.K. (2008). Mannan-coated gelatin nanoparticles for sustained and targeted delivery of didanosine: In vitro and in vivo evaluation. Acta Pharm..

[157] Yu W., Liu C., Liu Y., Zhang N., Xu W. (2010). Mannan-modified solid lipid nanoparticles for targeted gene delivery to alveolar macrophages. Pharm. Res..

[158] Zhan X., Jia L., Niu Y., Qi H., Chen X., Zhang Q., Zhang J., Wang Y., Dong L., Wang C. (2014). Targeted depletion of tumour-associated macrophages by an alendronate-glucomannan conjugate for cancer immunotherapy. Biomaterials.

[159] Zhu S., Niu M., O'Mary H., Cui Z. (2013). Targeting of tumor-associated macrophages made possible by PEG-sheddable, mannose-modified nanoparticles. Mol. Pharm..

[160] Niu M., Naguib Y.W., Aldayel A.M., Shi Y.C., Hursting S.D., Hersh M.A., Cui Z. (2014). Biodistribution and in vivo activities of tumor-associated macrophage-targeting nanoparticles incorporated with doxorubicin. Mol. Pharm..

[161] Niu M., Valdes S., Naguib Y.W., Hursting S.D., Cui Z. (2016). Tumor-associated macrophage-mediated targeted therapy of triple-negative breast cancer. Mol. Pharm..

[162] Ortega R.A., Barham W.J., Kumar B., Tikhomirov O., McFadden I.D., Yull F.E., Giorgio T.D. (2014). Biocompatible mannosylated endosomal-escape nanoparticles enhance selective delivery of short nucleotide sequences to tumor associated macrophages. Nanoscale.

[163] Ortega R.A., Barham W., Sharman K., Tikhomirov O., Giorgio T.D., Yull F.E. (2016). Manipulating the NF-κB pathway in macrophages using mannosylated, siRNA-delivering nanoparticles can induce immunostimulatory and tumor cytotoxic functions. Int. J. Nanomed..

[164] Yu S.S., Lau C.M., Barham W.J., Onishko H.M., Nelson C.E., Li H., Smith C.A., Yull F.E., Duvall C.L., Giorgio T.D. (2013). Macrophage-specific RNA interference targeting via “click”, mannosylated polymeric micelles. Mol. Pharm..

[165] Turk M.J., Waters D.J., Low P.S. (2004). Folate-conjugated liposomes preferentially target macrophages associated with ovarian carcinoma. Cancer Lett..

[166] Puig-Kroger A., Sierra-Filardi E., Dominguez-Soto A., Samaniego R., Corcuera M.T., Gomez-Aguado F., Ratnam M., Sanchez-Mateos P., Corbi A.L. (2009). Folate receptor β is expressed by tumor-associated macrophages and constitutes a marker for M2 anti-inflammatory/regulatory macrophages. Cancer Res..

[167] Nagai T., Tanaka M., Tsuneyoshi Y., Xu B., Michie S.A., Hasui K., Hirano H., Arita K., Matsuyama T. (2009). Targeting tumor-associated macrophages in an experimental glioma model with a recombinant immunotoxin to folate receptor β. Cancer Immunol. Immunother..

[168] Hattori Y., Yamashita J., Sakaida C., Kawano K., Yonemochi E. (2015). Evaluation of antitumor effect of zoledronic acid entrapped in folate-linked liposome for targeting to tumor-associated macrophages. J. Liposome Res..

[169] Daldrup-Link H.E., Golovko D., Ruffell B., Denardo D.G., Castaneda R., Ansari C., Rao J., Tikhomirov G.A., Wendland M.F., Corot C. (2011). MRI of tumor-associated macrophages with clinically applicable iron oxide nanoparticles. Clin. Cancer Res..

[170] Tang X. (2013). Tumor-associated macrophages as potential diagnostic and prognostic biomarkers in breast cancer. Cancer Lett..

[171] Fabriek B.O., Dijkstra C.D., van den Berg T.K. (2005). The macrophage scavenger receptor CD163. Immunobiology.

[172] Gordon S. (2003). Alternative activation of macrophages. Nat. Rev. Immunol..

[173] Etzerodt A., Maniecki M.B., Graversen J.H., Moller H.J., Torchilin V.P., Moestrup S.K. (2012). Efficient intracellular drug-targeting of macrophages using stealth liposomes directed to the hemoglobin scavenger receptor CD163. J. Control Release.

[174] Liu C., Sun C., Huang H., Janda K., Edgington T. (2003). Overexpression of legumain in tumors is significant for invasion/metastasis and a candidate enzymatic target for prodrug therapy. Cancer Res..

[175] Murthy R.V., Arbman G., Gao J., Roodman G.D., Sun X.F. (2005). Legumain expression in relation to clinicopathologic and biological variables in colorectal cancer. Clin. Cancer Res..

[176] Luo Y., Zhou H., Krueger J., Kaplan C., Lee S.H., Dolman C., Markowitz D., Wu W., Liu C., Reisfeld R.A. (2006). Targeting tumor-associated macrophages as a novel strategy against breast cancer. J. Clin. Investig..

[177] Liao D., Liu Z., Wrasidlo W., Chen T., Luo Y., Xiang R., Reisfeld R.A. (2011). Synthetic enzyme inhibitor: A novel targeting ligand for nanotherapeutic drug delivery inhibiting tumor growth without systemic toxicity. Nanomedicine.

[178] Zhang X., Tian W., Cai X., Wang X., Dang W., Tang H., Cao H., Wang L., Chen T. (2013). Hydrazinocurcumin Encapsuled nanoparticles “re-educate” tumor-associated macrophages and exhibit anti-tumor effects on breast cancer following STAT3 suppression. PLoS ONE.

[179] Song X., Wan Z., Chen T., Fu Y., Jiang K., Yi X., Ke H., Dong J., Yang L., Li L. (2016). Development of a multi-target peptide for potentiating chemotherapy by modulating tumor microenvironment. Biomaterials.

[180] Raes G., Brys L., Dahal B.K., Brandt J., Grooten J., Brombacher F., Vanham G., Noel W., Bogaert P., Boonefaes T. (2005). Macrophage galactose-type C-type lectins as novel markers for alternatively activated macrophages elicited by parasitic infections and allergic airway inflammation. J. Leukoc. Biol..

[181] Huang Z., Zhang Z., Jiang Y., Zhang D., Chen J., Dong L., Zhang J. (2012). Targeted delivery of oligonucleotides into tumor-associated macrophages for cancer immunotherapy. J. Control Release.

[182] Zhang M., Kim J.A. (2012). Effect of molecular size and modification pattern on the internalization of water soluble β-(1→3)-(1→4)-glucan by primary murine macrophages. Int. J. Biochem. Cell Biol..

[183] Zhang M., Gao Y., Caja K., Zhao B., Kim J.A. (2015). Non-viral nanoparticle delivers small interfering RNA to macrophages in vitro and in vivo. PLoS ONE.

[184] Cieslewicz M., Tang J., Yu J.L., Cao H., Zavaljevski M., Motoyama K., Lieber A., Raines E.W., Pun S.H. (2013). Targeted delivery of proapoptotic peptides to tumor-associated macrophages improves survival. Proc. Natl. Acad. Sci. USA.

[185] Cook J., Hagemann T. (2013). Tumour-associated macrophages and cancer. Curr. Opin. Pharmacol..

[186] Shmeeda H., Amitay Y., Tzemach D., Gorin J., Gabizon A. (2013). Liposome encapsulation of zoledronic acid results in major changes in tissue distribution and increase in toxicity. J. Control Release.

[187] Golbar H.M., Izawa T., Wijesundera K.K., Bondoc A., Tennakoon A.H., Kuwamura M., Yamate J. (2016). Depletion of hepatic macrophages aggravates liver lesions induced in rats by thioacetamide (TAA). Toxicol. Pathol..

[188] Liu X., Liu Y., Zhao L., Zeng Z., Xiao W., Chen P. (2017). Macrophage depletion impairs skeletal muscle regeneration: The roles of regulatory factors for muscle regeneration. Cell Biol. Int..

[189] Mosser D.M., Edwards J.P. (2008). Exploring the full spectrum of macrophage activation. Nat. Rev. Immunol..

[190] Patel S.K., Janjic J.M. (2015). Macrophage targeted theranostics as personalized nanomedicine strategies for inflammatory diseases. Theranostics.

[191] Hoppstadter J., Seif M., Dembek A., Cavelius C., Huwer H., Kraegeloh A., Kiemer A.K. (2015). M2 polarization enhances silica nanoparticle uptake by macrophages. Front. Pharmacol..

